# A model-based clustering via mixture of hierarchical models with covariate adjustment for detecting differentially expressed genes from paired design

**DOI:** 10.1186/s12859-023-05556-x

**Published:** 2023-11-08

**Authors:** Yixin Zhang, Wei Liu, Weiliang Qiu

**Affiliations:** 1 School of Mathematical Science, University of Science and Technology of China, Hefei, Anhui China; 2 Department of Mathematics and Statistics, York University, Toronto, ON Canada; 3 Department of Biostatistics and Programming, Sanofi, Cambridge, MA USA

**Keywords:** Curse of dimensionality, Confounding, EM algorithm, RNAseq

## Abstract

The causes of many complex human diseases are still largely unknown. Genetics plays an important role in uncovering the molecular mechanisms of complex human diseases. A key step to characterize the genetics of a complex human disease is to unbiasedly identify disease-associated gene transcripts on a whole-genome scale. Confounding factors could cause false positives. Paired design, such as measuring gene expression before and after treatment for the same subject, can reduce the effect of known confounding factors. However, not all known confounding factors can be controlled in a paired/match design. Model-based clustering, such as mixtures of hierarchical models, has been proposed to detect gene transcripts differentially expressed between paired samples. To the best of our knowledge, no model-based gene clustering methods have the capacity to adjust for the effects of covariates yet. In this article, we proposed a novel mixture of hierarchical models with covariate adjustment in identifying differentially expressed transcripts using high-throughput whole-genome data from paired design. Both simulation study and real data analysis show the good performance of the proposed method.

**Supplementary Information:**

The online version contains supplementary material available at 10.1186/s12859-023-05556-x.

## Introduction

Genome-wide differential gene expression analysis is widely used for the elucidation of the molecular mechanisms of complex human diseases. One popular and powerful approach to detect differentially expressed genes is the probe-wise linear regression analysis combined with the control of multiple testing, such as limma [1]. That is, we first perform linear regression for each probe and then adjust p-values for controlling multiple testing. One advantage of this approach is its capacity to adjust for potential confounding factors.

Another approach for detecting differentially expressed genes is the model-based clustering via mixture of Bayesian hierarchical models (MBHM) [2–7], which can borrow information across genes to cluster genes. Probe clustering based on MBHMs treats gene transcripts as “samples” and samples as “variables”. Therefore, transcript clustering based on MBHMs has large number of “samples” and relatively small number of “variables”, hence does not have the curse-of dimensionality problem. In addition, unlike transcript-specific tests that have several parameters per transcript, transcript clustering based on MBHMs has only a few hyperparameters per cluster to be estimated and could borrow information across transcripts to estimate model hyperparameters. These approaches generally assume that samples under two groups are obtained independently. [8] proposed a constrained MBHM to identify genetic outcomes measured from paired/matched designs.

Paired design is commonly used in study design for its homogeneous external environment for comparing measurements under different conditions. However, not all known confounding factors can be controlled in a paired/match design. Hence, we might still need to adjust the effects of confounding factors for data from a paired/matched design.

Mixture of regressions or mixture of experts model [9–11] have been proposed in literature to do clustering with capacity to adjust for covariates. To best of our knowledge, this approach does not have constraints on positive, negative, and constant means and has not been applied to detect differentially expressed genes.

In this article, we proposed a novel mixture of hierarchical models with covariate adjustment in identifying differentially expressed transcripts using high-throughput whole genome data from paired design.

## Method

We assumed that gene transcripts can be roughly classified into 3 clusters based on their expression levels in subjects after treatment (denoted as condition 1) relative to those before treatment (denoted as condition 2):

1. Transcripts after treatment have higher expression levels than those before treatment, i.e., over-expressed (**OE**) in condition 1;
2. Transcripts after treatment have lower expression levels than those before treatment, i.e., under-expressed (**UE**) in condition 1;
3. Transcripts after treatment have same expression levels than those before treatment, i.e., non-differentially expressed (**NE**) between condition 1 and matched condition 2.

We followed [8] to directly model the marginal distributions of gene transcripts in the 3 clusters. In [8], they proposed a mixture of three-component hierarchical distributions to characterize the within-pair difference of gene expression. We extended their model by incorporating potential confounding factors (such as *Age* and *Sex*) in the mixture of hierarchical models, which might affect the response of gene expression to drug treatment.

Note that this extension is non-trivial, just like multiple linear regression is not just a simple extension to simple linear regression.

We assumed that data have been processed so that the distributions of mRNA expression levels are close to normal distributions. For RNAseq data, we can apply VOOM transformation [12] or countTransformers [13] before applying *eLNNpairedCov*.

### A mixture of hierarchical models

For the $g^{th}$ gene transcript, let $x_{gl}$ and $y_{gl}$ denote the expression levels of the $l^{th}$ subject under two different conditions, e.g., before and after treatment, $g=1, \ldots , G$, $l=1, \ldots , n$, where *G* is the number of transcripts and *n* is the number of subjects (i.e., the number of pairs). Let $d_{gl} = \log _2(y_{gl}) - \log _2(x_{gl})$ be the log2 difference for the $g^{th}$ gene transcript of $l^{th}$ subject. Denote ${{\textbf {d}}}_{g}=\left( d_{g1}, \ldots , d_{gn}\right) ^T$. We assumed that ${\textbf {d}}_{g}$ is conditionally normally distributed given mean vector and covariance matrix. Let ${\varvec{W}}^{T}$ be the $n\times (p+1)$ design matrix, where *p* is the number of covariates. The first column of ${\varvec{W}}^T$ is the vector of ones, indicating intercept. Let ${\varvec{\eta }}$ be the $(p+1)\times 1$ vector of coefficients for the intercept and covariate effects. We assume following mixture of three-component hierarchical models:

For gene transcripts over-expressed (OE) in post-treatment samples, we expect that the mean log2 differences are positive. Hence, we assume

$$\begin{aligned}{} & {} {\textbf {d}}_g|\left( {\varvec{\mu }}_g, \tau _g \right) \sim N\left( {\varvec{\mu }}_g, \tau _g^{-1}{\varvec{I}}_n\right) \\{} & {} \quad {\varvec{\mu }}_g|\tau _g \sim N\left( \exp [{\varvec{W}}^T{\varvec{\eta }}_1], k_1\tau _g^{-1} {\varvec{I}}_n \right) \\{} & {} \quad \tau _g \sim \Gamma \left( \alpha _1,\beta _1\right) \end{aligned}$$

where $k_1>0, \alpha _1>0$ and $\beta _1>0$. $\Gamma \left( \alpha _1, \beta _1\right)$ denotes the Gamma distribution with shape parameter $\alpha _1$ and rate parameter $\beta _1$. That is, we assume that (1) the mean vectors ${\varvec{\mu }}_g$, $g=1$, $\ldots$, *G*, given the variance $\tau _g^{-1}$ follow a multivariate normal distribution with mean vector $\exp \left[ {\varvec{W}}^T{\varvec{\eta }}_1\right]$ and covariance matrix $k_1 \tau _g^{-1} {\varvec{I}}_n$; and (2) the variances $\tau _g^{-1}$, $g=1$, $\ldots$, *G*, follow a Gamma distribution with shape parameter $\alpha _1$ and rate parameter $\beta _1$.

Note that the exponential of the intercept $\exp (\eta _{10})$ indicates the mean of log2 difference is positive.

For gene transcripts under-expressed (UE) in post-treatment samples, we expect that the mean log2 differences are negative. Hence, we assume

$$\begin{aligned}{} & {} {\textbf {d}}_g|\left( {\varvec{\mu }}_g, \tau _g \right) \sim N\left( {\varvec{\mu }}_g, \tau _g^{-1}{\varvec{I}}_n\right) \\{} & {} \quad {\varvec{\mu }}_g|\tau _g \sim N\left( -\exp [{\varvec{W}}^T{\varvec{\eta }}_2], k_2 \tau _g^{-1} {\varvec{I}}_n\right) \\{} & {} \quad \tau _g \sim \Gamma \left( \alpha _2,\beta _2\right) \end{aligned}$$

where $k_2>0, \alpha _2>0$, $\beta _2>0$, and ${\varvec{W}}^T$ is the design matrix.

Note that the negative exponential of the intercept $-\exp (\eta _{20})$ indicates the mean of log2 difference is negative.

For gene transcripts non-differentially expressed (NE) between pre- and post-treatment samples, we expect the mean log2 differences are zero. Hence, we assume

$$\begin{aligned}{} & {} {\textbf {d}}_g| \tau _g \sim N\left( {\varvec{U}}^T {\varvec{\theta }}_g, \tau _g^{-1}{\varvec{I}}_n\right) \\{} & {} \quad {\varvec{\theta }}_g| \tau _g \sim N({\varvec{\eta }}_3, k_3\tau _g^{-1} {\varvec{I}}_p)\\{} & {} \quad \tau _g \sim \Gamma \left( \alpha _3,\beta _3\right) \end{aligned}$$

where $k_3>0, \alpha _3>0$ and $\beta _3>0$. ${\varvec{U}}^T$ is the design matrix without intercept column. That is, the intercepts are zero. Note that the intercepts indicate mean log2 differences. Hence, ${\varvec{\eta }}_3$ is a $p\times 1$ vector of coefficients for the covariates.

Note that ${\varvec{\theta }}_g$ measure effects of confounding factors for NE genes. The true effect of NE genes are zero (i.e., the intercept of ${\varvec{U}}^T {\varvec{\theta }}_g$ is zero in the above model).

The hyperparameters $\alpha _c$ and $\beta _c$ are shape and rate parameters for the Gamma distribution, respectively, $c = 1,2,3$. As for $k_1, k_2$ and $k_3$, the variation of the mean vector ${\varvec{\mu }}_g$ should be smaller than that of the observations ${\textbf {d}}_g$. So we expect $0< k_c < 1, c=1,2,3$.

Note that the marginal distribution for each component of the mixture is a multivariate *t* distribution [14, Section 3.7.6]. However, to model differentially expressed genes, the multivariate *t* distributions derived from our models have special structure of mean vector and covariance matrix.

For continuous covariates, we require that they are standardized so that they have mean zero and variance one. Standardizing continuous covariates would make $\exp \left( {\varvec{W}}^T{\varvec{\eta }}_1\right)$ and $\exp \left( {\varvec{W}}^T{\varvec{\eta }}_2\right)$ be numerically finite.

Ideally, we should require ${\varvec{\mu }}_g > 0$ $( {\varvec{\mu }}_g < 0)$ for all transcripts in cluster 1 (cluster 2). To do so, we can assume a log normal prior distribution for ${\varvec{\mu }}_g$ in cluster 1, for instance. However, a log normal distribution could not be a conjugate prior for the mean of a normal distribution. It would increase the computational burden if non-conjugate priors were used. Other alternative models can also be used, such as assuming ${\varvec{\mu }}_g | \eta _{10}= \exp (\eta _{10}) + {\varvec{W}}^T{\varvec{\eta }}_1$ and $\eta _{10}$ follows a normal distribution. However, these models do not have closed-form marginal densities. Hence, they would substantially increase computational burden. Besides, the empirical distribution of the mean log2 difference ${\textbf {d}}_{g}$ of the differentially expressed gene probes has shown a right-skewed pattern, while that of non-differentially expressed genes demonstrates an approximate bell shape (see in Additional file 1: Figures A2-A4). Hence, we require the mean $E({\varvec{\mu }}_g)>0$ ($E({\varvec{\mu }}_g)<0$) for cluster 1 (cluster 2) by assuming $E({\varvec{\mu }}_g)$ for cluster 1 (cluster 2) to be $\exp [{\varvec{W}}^T{\varvec{\eta }}_1]$ ($-\exp [{\varvec{W}}^T{\varvec{\eta }}_2]$).

The proposed mixture models have meaningful biological interpretations for mean structures. In particular, for the **OE** cluster, the intercept $\exp (\eta _{10})$ can be interpreted as the expected average log2 difference of gene transcripts when the value of all the *p* covariates are zero; the coefficient $\eta _{1i}$ of covariate *i* can be interpreted as there exists $\exp (\eta _{1i})$ fold-change associated with the one unit increase in covariate *i* while the values of the remaining $( p-1 )$ covariates are fixed; for the **UE** cluster, the intercept $-\exp (\eta _{20})$ can be interpreted as the expected average log2 difference of gene transcripts when the value of all the *p* covariates are zero; the coefficient $\eta _{2i}$ of covariate *i* can be interpreted as there exists $\exp (\eta _{2i})$ fold-change associated with the one unit increase in covariate *i* while the values of the remaining $(p-1)$ covariates are fixed; while for the **NE** cluster, the coefficient $\eta _{3i}$ of covariate *i* can be interpreted as $\eta _{3i}$ unit increase of expected log2 difference of gene transcripts associated with the one unit increase in covariate *i* while the values of the remaining $(p-1)$ covariates are fixed. They also are convenient to get closed-form marginal densities so that we can use Expectation-Maximization (EM) algorithm to estimate hyperparameters, instead of using computational-intensive algorithms, such as Markov chain Monte Carlo (MCMC).

### Marginal density functions

Let $f_1(\textbf{d}_g|{\psi })$, $f_2(\textbf{d}_g|{\psi })$, $f_3(\textbf{d}_g|{\psi })$ be the marginal densities of the 3 hierarchical models, and $\varvec{\pi }$ $=$ $(\pi _1,\pi _2,\pi _3)$ be the vector of cluster mixture proportions, where ${\psi }=\left( \alpha _1, \beta _1, k_1, {\varvec{\eta }}_1^T, \alpha _2, \beta _2, k_2, {\varvec{\eta }}_2^T, \alpha _3, \beta _3, k_3, {\varvec{\eta }}_3^T\right) ^T$. Then the marginal density of ${\textbf {d}}_g$ is:

$$\begin{aligned} f({\textbf{d}}_g|\psi )=\pi _1f_1(\textbf{d}_g|{\psi })+\pi _2f_2(\textbf{d}_g|{\psi })+\pi _3f_3(\textbf{d}_g|\psi ). \end{aligned}$$

### Determining transcript cluster membership

The transcript-cluster membership is determined based on the posterior probabilities, $\zeta _{gc}$ $=$ $Pr(g^{th}$ gene transcript in cluster *c* $|{\varvec{d}}_g)$. We can get

1 $$\begin{aligned} \begin{aligned} \zeta _{gc} = \dfrac{\pi _cf_c(\textbf{d}_g|{\psi })}{\pi _1f_1(\textbf{d}_g|{\psi })+\pi _2f_2(\textbf{d}_g|{\psi })+\pi _3f_3(\textbf{d}_g|\psi )}, c=1,2,3. \end{aligned} \end{aligned}$$

We determine a transcript’s cluster membership as follows: If the maximum value among $\zeta _{gi}, i=1,2,3$ is $\zeta _{gc}$, then the transcript *g* belongs to cluster *c*.

The true values of $\pi _1$, $\pi _2$, $\pi _3$, and ${\psi }$ are unknown. We use estimated values to determine transcripts’ cluster membership.

### Parameter estimation via EM algorithm

We used expectation-maximization (EM) algorithm [15] to estimate the model parameters ${\varvec{\pi }}=\left( \pi _1, \pi _2, \pi _3\right) ^T$ and ${\psi }$.

Let ${\varvec{z}}_g = (z_{g1}, z_{g2}, z_{g3})$ to be the indicator vector indicating if gene transcript *g* belongs to a cluster or not. To stablize the estimate of ${\varvec{\pi }}$ when $\pi _c$ is very small, we assume that the cluster mixture proportions $\varvec{\pi }$ follows a symmetric Dirichlet $D(\textbf{b})$ distribution, i.e.,$f(\varvec{\pi })=\frac{\Gamma (\sum _{c=1}^{3}b_c)}{\prod _{c=1}^{3}\Gamma (b_c)}\prod _{c=1}^{3}\pi _c^{b_c-1}$. Therefore, the likelihood function for the complete data $(\textbf{d}, \varvec{z}, \varvec{\pi })$ is

$$\begin{aligned} L({\psi }|\textbf{d}, \varvec{z}, \varvec{ \pi }) =&\,f(\textbf{d}, \varvec{z}, \varvec{\pi } | {\psi })\\ = &\,f(\textbf{d} , \varvec{z}| \varvec{\pi }, {\psi }) f(\varvec{\pi } | {\psi })\\ = &\,f(\textbf{d}, \varvec{z}| \varvec{\pi }, {\psi }) f(\varvec{\pi } )\\ = &\left( \prod _{g=1}^{G} f(\textbf{d}_g, \varvec{z}_g | \psi ,\varvec{\pi })\right) Dir(\textbf{b})\\ = &\left( \prod _{g=1}^{G}(\pi _1f_1(\textbf{d}_g|{\psi }))^{z_{g1}}(\pi _2f_2(\textbf{d}_g|{\psi })) ^{z_{g2}}(\pi _3f_3(\textbf{d}_g|{\psi }))^{z_{g3}}\right) \\&\times \frac{\Gamma (\sum _{c=1}^{3}b_c)}{\prod _{c=1}^{3}\Gamma (b_c)}\prod _{c=1}^{3}\pi _c^{b_c-1}. \end{aligned}$$

Then the log complete-data likelihood function is:

$$\begin{aligned} l({\psi }|\textbf{d}, \varvec{z}, \varvec{\pi })&= \sum _{g=1}^{G}((z_{g1}\log f_1(\textbf{d}_g|{\psi })+z_{g2} \log f_2(\textbf{d}_g|{\psi }))+z_{g3}\log f_3(\textbf{d}_g|{\psi })) \\&+ \sum _{g=1}^{G}(z_{g1}\log \pi _1+z_{g2}\log \pi _2+z_{g3}\log \pi _3)\\&+\log \left( \frac{\Gamma (\sum _{c=1}^{3}b_c)}{\prod _{c=1}^ {3}\Gamma (b_c)}\right) +\sum _{c=1}^{3}(b_c-1)\log \pi _c. \end{aligned}$$

The EM algorithm is used to estimate parameters ${\varvec{\pi }}$ and ${\psi }$. Since ${\varvec{z}}$ is unknown random vector, we integrate it out from the log complete-data likelhood function. Here, $\varvec{z}_g=(z_{g1},z_{g2},z_{g3})$.

2 $$\begin{aligned} \begin{aligned} \zeta _{g1}&=E(z_{g1}|\textbf{d}_g,\varvec{\pi },\psi )\\&=Pr(z_{g1}=1|\textbf{d}_g,\varvec{\pi },{\psi })\\&=\dfrac{\pi _1f_1({\textbf{d}}_g|{\psi })}{\pi _1f_1(\textbf{d}_g|{\psi })+\pi _2f_2(\textbf{d}_g|{\psi })+\pi _3f_3(\textbf{d}_g|\psi )}\\ \zeta _{g2}&=\dfrac{\pi _2f_2({\textbf{d}}_g|{\psi })}{\pi _1f_1(\textbf{d}_g|{\psi })+\pi _2f_2(\textbf{d}_g|{\psi })+\pi _3f_3(\textbf{d}_g|\psi )}\\ \zeta _{g3}&=\dfrac{\pi _3f_3({\textbf{d}}_g|{\psi })}{\pi _1f_1(\textbf{d}_g|{\psi })+\pi _2f_2(\textbf{d}_g|{\psi })+\pi _3f_3(\textbf{d}_g|\psi )} \end{aligned} \end{aligned}$$

**E-step**. Denote $Q^{(t)}\left( \varvec{\pi }, {\psi } | {\textbf {d}}, {\varvec{z}}^{(t)}, \varvec{\pi }^{(t)}\right)$ as the expected log complete-data likelihood function at *t*-th iteration of the EM algorithm, we have

$$\begin{aligned} Q^{(t)}&= E_{{\varvec{z}}}\left[ l\left( {\psi }\left| {\textbf {d}}, {\varvec{z}}, \varvec{\pi } \right. \right) | {\varvec{d}}, {\varvec{z}}^{(t)}, \varvec{\pi }^{(t)}\right] \\&= \sum _{g=1}^{G}( ( \zeta _{g1}^{(t)}\log f_1(\textbf{d}_g|{\psi })+ \zeta _{g2}^{(t)}\log f_2(\textbf{d}_g|{\psi }))+ \zeta _{g3}^{(t)}\log f_3(\textbf{d}_g|{\psi })) \\&+ \sum _{g=1}^{G}(\zeta _{g1}^{(t)}\log \pi _1+\zeta _{g2}^{(t)}\log \pi _2+\zeta _{g3}^{(t)}\log \pi _3)\\&+\log \left( \frac{\Gamma (\sum _{c=1}^{3}b_c)}{\prod _{c=1}^{3}\Gamma (b_c)}\right) +\sum _{c=1}^{3}(b_c-1)\log \pi _c, \end{aligned}$$

where

3 $$\begin{aligned} \begin{aligned} \zeta _{gc}^{(t)}&=E\left( z_{gc}|\textbf{d}_g,\varvec{\pi }^{(t)}, {\psi }^{(t)}\right) \\&=\dfrac{\pi _c^{(t)} f_c(\textbf{d}_g|{\psi }^{(t)})}{\pi _1^{(t)} f_1(\textbf{d}_g|{\psi }^{(t)})+\pi _2^{(t)} f_2(\textbf{d}_g|{\psi }^{(t)})+\pi _3^{(t)} f_3(\textbf{d}_g|\psi ^{(t)})}, \ c = 1,2,3. \end{aligned} \end{aligned}$$

**M-step**. Maximize $Q^{(t)}\left( \varvec{\pi }, {\psi } | {\textbf {d}}, {\varvec{z}}^{(t)}, \varvec{\pi }^{(t)}\right)$ to find the optimal values of ${\varvec{\pi }}$ and ${\psi }$, and use these optimal values as estimates for the parameters ${\varvec{\pi }}$ and ${\psi }$.

To maximize $Q^{(t)}\left( \varvec{\pi }, {\psi } | \textbf{d}, {\varvec{z}}^{(t)}, \varvec{\pi }^{(t)}\right)$, we use the “L-BFGS-B” method developed by Byrd et al. (1995) [16], which utilizes the first partial derivatives of $Q^{(t)}\left( \varvec{\pi }, {\psi } | {\textbf {d}}, {\varvec{z}}^{(t)}, \varvec{\pi }^{(t)}\right)$ and allows box constraints, that is each variable can be given a lower and/or upper bound.

#### Simulated annealing modification

EM algorithm may be trapped in a local maximum since it is strictly ascending. As introduced by Celeux and Govaert (1992) [17], simulated annealing (SA) is widely used to help EM algorithm escape from local maximum by adding randomness with a stochastic step. Specifically, the conditional expectation in (2) is modified in a SA algorithm as follows

4 $$\begin{aligned} \begin{aligned} {\tilde{\zeta }}_{gc}^{(t)} = \dfrac{ \left[ \pi _c ^{(t)}f_c(\textbf{d}_g|{\psi }^{(t)}) \right] ^{1/m^{(t)}} }{ \sum _{c=1}^{3} \left[ \pi _c^{(t)}f_c({\textbf{d}}_g|{\psi }^{(t)}) \right] ^{1/m^{(t)}} },\ c=1,2,3. \end{aligned} \end{aligned}$$

where *m* is the temperature used to control the randomness. Usually, the temperature *m* starts with a relatively high value since larger *m* leads to larger randomness. At iteration *t*, the temperature is updated by $m^{(t+1)} = r\times m^{(t)}$ with the cooling rate *r* controls the speed of reduction. As suggested in [18, 19], we use $m^{(0)}=2$ and $r= 0.9$.

We denoted *eLNNpairedCov* as the proposed method using the traditional EM algorithm to obtain parameter estimates and denoted *eLNNpairedCov.SEM* as the proposed method using the EM with SA-modification to obtain parameter estimates.

We stop the expectation-maximization iterations based on a proportional change, i.e. if the maximum of the absolute value of the differences of model parameter estimates between current iteration and previous iteration over the absolute value of the previous iteration estimates is smaller than a small constant (e.g. $1.0\times 10^{-3}$).

More details about the EM algorithm are shown in Supplementary Document [see Additional file 1].

### A real data study

We used the dataset GSE24742 [20], which can be downloaded from the Gene Expression Omnibus [https://www.ncbi.nlm.nih.gov/geo/query/acc.cgi?acc=GSE24742], to evaluate the performance of the proposed model-based clustering methods (denoted as *eLNNpairedCov* and *eLNNpairedCov.SEM* ).

The dataset is from a study that investigated the gene expression before and after administrating rituximab, a drug for treating anti-TNF resistant rheumatoid arthritis (RA). There are 12 subjects, each having 2 samples (one sample is before treatment and the other is after treatment). Age and sex are also available. Expression levels of 54,675 gene probes were measured for each of the 24 samples by using Affymetrix HUman Genome U133 Plus 2.0 array. The dataset has been preprocessed by the dataset contributor. We further kept only 43,505 gene probes in the autosomal chromosomes (i.e., chromosomes 1 to 22). We then performed log2 transformation for gene expression levels. We next obtained the within-subject difference of the log2 transformed expression levels (log2 expression after-treatment minus log2 expression before-treatment). By examining the histogram (Figure A1) [see Additional file 1] of the estimated standard deviations of log2 differences of within-subject gene expression for the 43,505 gene probes, we found a bimodal distribution. Based on Figure A1 [see Additional file 1], where the histogram of estimated standard deviations exhibits two modes, we choose to exclude gene probes with standard deviation $<1$ corresponding to the first mode. It is a common practice to remove genes with low variation [21–23]. Finally, 23,948 gene probes kept in the down-stream analysis.

### A simulation study

We performed a simulation study to compare the performance of the proposed methods *eLNNpairedCov*, *eLNNpairedCov.SEM* with transcript-wise test *limma* and Li et al.’s [8] method (denoted as *eLNNpaired*). *eLNNpairedCov*, *eLNNpairedCov.SEM* and *limma* adjust covariate effects, while *eLNNpaired* does not. For *eLNNpaired*, we first regress out covariates effect for each gene to make a fair comparison between *eLNNpaired* and other methods.

The *limma* approach first performs an empirical-Bayes-based linear regression for each transcript. In this linear regression, the within-subject log2 difference of transcript expression is the outcome and intercept indicating if the transcript is over-expressed (intercept>0), under-expressed (intercept<0), or non-differentially expressed (intercept = 0), adjusting for potential confounding factors. A transcript is claimed as OE if its intercept estimate is positive and corresponding FDR-adjusted p-value $<0.05$, where FDR stands for false discovery rate. A transcript is claimed as UE if its intercept estimate is negative and corresponding FDR-adjusted p-value $<0.05$. Other transcripts are claimed as NE.

The parameter values (${\varvec{\pi }}$, ${\psi }$, and proportion of women) in the simulation study are based on the estimates via *eLNNpairedCov.SEM* from the analysis of the pre-processed real dataset GSE24742 described in Subsection “*A real data study*”.

In this simulation study, we considered two sets with different covariate coefficients for differentially expressed genes clusters. In the first set (Set 1), parameter values are the estimates of parameters based on the *eLNNpairedCov.SEM* method from real dataset. That is, $\pi _1=0.00246$, $\pi _2 = 0.01470$, $\pi _3 = 0.98284$, $\alpha _1 = 3.53$, $\beta _1=3.45$, $k_1=0.26$, $\eta _{10}=0.18$, $\eta _{11} = 0.00$, $\eta _{12} = -1.05$, $\alpha _2 = 3.53$, $\beta _2 = 3.45$, $k_2=0.26$, $\eta _{20} = 0.18$, $\eta _{21} = 0.00$, $\eta _{22} = -1.05$, $\alpha _3 = 2.86$, $\beta _3=2.20$, $k_3 = 0.72$, $\eta _{31} = -0.01$, $\eta _{32} = 0.00$. In the second set (Set 2), we set $\eta _{10}$=$\eta _{20}$=0.08 instead of 0.18. For each set, we considered two scenarios. In the first scenario (Scenario1), the number of subjects is equal to 30. In the second scenario (Scenario2), the number of subjects is equal to 100.

For each scenario, we generated 100 datasets. Each simulated dataset contains $G=20,000$ gene transcripts. There are two covariates: standardized age (denoted as *Age.s*) and *Sex*. *Age.s* follows normal distribution with mean 0 and standard deviation 1. Seventy five percent (75%) of subjects are women.

### Evaluation criteria

Two agreement indices and two error rates are used to compare the predicted cluster membership and true cluster membership of all genes. The two agreement indices are accuracy (i.e., proportion of predicted cluster membership equal to the true cluster membership) and Jaccard index [24]. For perfect agreement, these indices have a value of one. If an index takes a value close to zero, then the agreement between the true transcript cluster membership and the estimated transcript cluster membership is likely due to chance. The two error rates are false positive rate (FPR) and false negative rate (FNR). FPR is the percentage of detected DE transcripts among truly NE transcripts. FNR is the percentage of detected NE transcripts among truly DE transcripts. We also examined the user time and number of EM iterations for running each simulated dataset.

## Results

### Results of the real data analysis

For the real dataset, we adjusted standardized age and sex for *eLNNpairedCov*, *eLNNpairedCov.SEM*, and *limma*. We standardized age so that it has mean zero and variance one. For each transcript, we also scaled its expression across subjects so that its variance is equal one. For *eLNNpaired*, we first regressed out the effect of standardized age and sex for each transcript.

The estimates of parameters in our model are listed in Table 1. Note that the proposed *eLNNpairedCov* and *eLNNpairedCov.SEM* have the same estimates for the parameters in these three clusters, except for the proportions of three clusters. The proportions of OE and UE estimated by *eLNNpairedCov* method are 0.0376% and 0.346%, respectively. The proportions of OE and UE estimated by *eLNNpairedCov.SEM* method are 0.246% and 1.47%, respectively.

**Table 1:** Parameter estimates of OE, UE and NE clusters from *eLNNpairedCov* and *eLNNpairedCov.SEM*

OE		UE		NE	
$\beta _1$	3.445543	$\beta _2$	3.445543	$\beta _3$	3.445543
$k_1$	0.264565	$k_2$	0.264565	$k_3$	0.264565
$\eta _{10}$	0.176007	$\eta _{20}$	0.176007		
$\eta _{11}$	−0.000609	$\eta _{21}$	−0.000609	$\eta _{31}$	−0.013796
$\eta _{12}$	−1.051257	$\eta _{22}$	−1.051257	$\eta _{32}$	−0.000017

For the OE cluster, $\exp (\eta _{10})=\exp (0.176007)=1.192$ can be interpreted as the expected log2 difference for a male subject ($sex=0$) whose age is equal to mean age ($age=0$ is the mean-centered age); $\eta _{11}=-0.000609$ indicates that one-unit increase in *age* leads to $\exp (-0.000609)=0.999$ fold-changes in expected log2 difference, while $\eta _{12}=-1.051257$ indicates that there is $\exp (-1.051257)=0.349$ fold-changes between male subjects and female subjects in expected log2 difference if they are at the same *age*. For the UE cluster, $\eta _{20}=0.176007$ can be interpreted as the expected log2 difference for a male subject ($sex=0$) whose age is equal to mean age ($age=0$ is the mean-centered age) is $-\exp (0.176007)=-1.192$; $\eta _{21}=-0.000609$ indicates that one-unit increase in *age* leads to $\exp (-0.000609)=0.999$ fold-changes in expected log2 difference, while $\eta _{22}=-1.051257$ indicates that there is $\exp (-1.051257)=0.349$ fold-changes between male subjects and female subjects in expected log2 difference if they are at the same *age*. For the NE cluster, $\eta _{31}=-0.013796$ indicates that one-unit increase in *age* leads to 0.01379 decreases in expected log2 difference, and $\eta _{32}=-0.000017$ indicates that there is 0.000017 decrease from female subjects to male subjects in the expected log2 difference if they are at the same *age*.

The number of differentially expressed genes detected by each method is listed in Table 2.

**Fig. 1:**
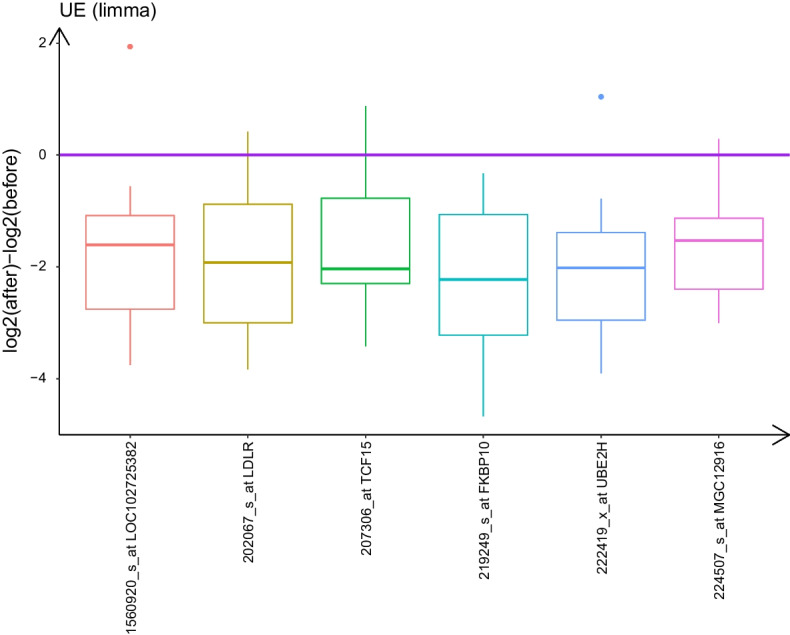
Parallel boxplots of log2 within-subject difference of gene expression for 6 UE transcripts detected by *limma* for pre-processed GSE24742 dataset. Red horizontal line indicates log2 difference equal to zero

**Fig. 2:**
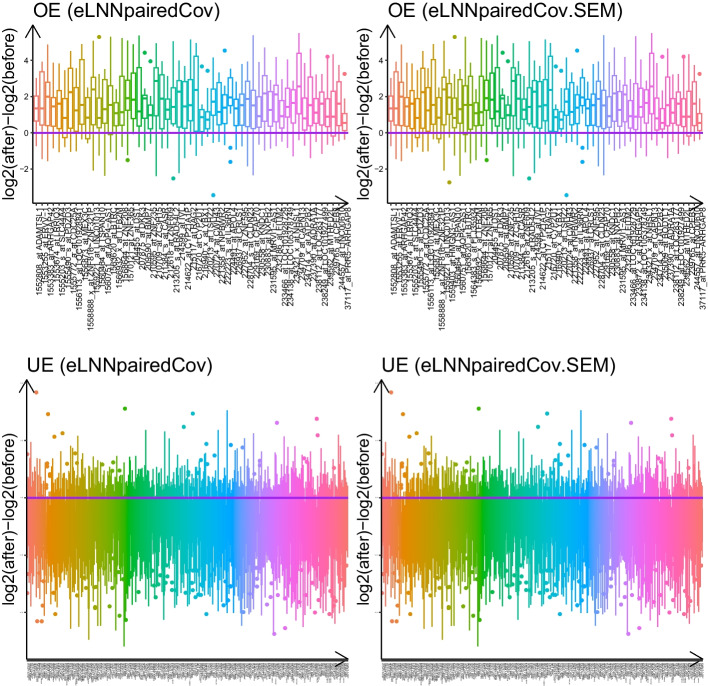
Parallel boxplots of log2 within-subject difference of gene expression for differentially expressed transcripts detected by *eLNNpairedCov* and *eLNNpairedCov.SEM* for pre-processed GSE24742 dataset. Upper two panels: 55 OE transcripts and 59 OE transcripts, respectively; Lower two panels: 355 UE transcripts and 352 UE transcripts, respectively. Red horizontal lines indicate log2 difference equal to zero

The *limma* method detected 6 under-expressed gene transcripts (Figure 1 and Table S1), while *eLNNpaired* did not find any positive signals (i.e., ${\hat{\pi }}_3 = 1$). The proposed methods *eLNNpairedCov* and *eLNNpairedCov.SEM* detected 55 OE transcripts (Table S2) and 59 OE transcripts (Table S3), respectively (Upper two panels of Fig. 2) and 355 UE transcripts (Table S4) and 352 UE transcripts (Table S5), respectively (Lower two panels of Figure 2). The 6 UE transcripts detected by *limma* is also selected as UE transcripts by *eLNNpairedCov* and *eLNNpairedCov.SEM*. Note that the 55 OE genes detected by *eLNNpairedCov* are also detected by *eLNNpariedCov.SEM*. The 352 UE genes detected by *eLNNpairedCov.SEM* are also detected by *eLNNpariedCov*.

**Table 2:** Number of Differentially expressed genes detected by *limma*, *eLNNpaired*, *eLNNpairedCov* and *eLNNpairedCov.SEM* in GSE24742

	limma	eLNNpaired	eLNNpairedCov	eLNNpairedCov.SEM
OE	0	0	55	59
UE	6	0	355	352

It is assuring that several genes corresponding to the DE transcripts identified by *eLNNpairedCov* and *eLNNpairedCov.SEM* have been associated to rheumatoid arthritis (RA) in literature. For example, Humby et al. (2019) [25] reported that genes *ZNF365* (OE), *IL36RN* (OE), *MRVI1-AS1* (OE), *WFDC6 * (UE), *UBE2H* (UE), are associated with RA.

We performed pathway enrichment analysis through the use of IPA (QIAGEN Inc., https://www.qiagenbioinformatics.com/products/ingenuitypathway-analysis) for 352 UE and 55 OE genes identified by *eLNNpairedCov.SEM*. The top enriched canonical pathways are shown in Tables 3 and 4. Evidence in literature shows that these pathways are relevant to RA. S100 protein family plays an important role in rheumatoid arthritis ( [26]). Literature shows consistent crucial role of the PD-1/PD-L pathway in the pathogenesis of rheumatic diseases ( [27, 28]). It has been shown that RA can lead to lung tissue damage, resulting in pulmonary fibrosis ( [29]). Macrophage is a key player in the pathogenesis of autoimmune diseases, such as RA ( [30]). RA and osteoarthritis (OA) are two common arthritis with different pathogenesis ( [31]). It is interesting to see Osteoarthritis pathway is a significantly enriched pathway for UE genes. It is consistent with literature that similar focal and systemic alterations exist in RA and OA [32].

Ribonucleotide Reductase (RNR) is the enzyme providing the precursors needed for both synthesis and repair of DNA, which could be a potential drug for RA ( [33, 34]). Leukocyte extravasation through the endothelial barrier is important in the pathogenesis of RA ( [35]). It has been shown that the limb bud and heart development (LBH) gene is a key dysregulated gene in RA and other autoimmune diseases and there are some evidence showing LBH could modulate the cell cycle [36]. Osteoblasts, osteoclasts and chondrocytes play importan roles in Rheumatoid Arthritis ( [37–39]). We did not find literature linking Tetrahydrofolate Salvage from 5,10- methenyltetrahydrofolate to RA yet, indicating this enrichment might be novel.

**Table 3:** Top canonical pathways for 352 UE genes by *eLNNpairedCov.SEM*

Name	p-value
S100 Family Signaling Pathway	$2.97E-06$
PD-1, PD-L1 cancer immunotherapy pathway	$7.54E-05$
Pulmonary Fibrosis Idiopathic Signaling pathway	$3.45E-04$
Phagosome Formation	$7.56E-04$
Osteoarthritis Pathway	$1.04E-03$

**Table 4:** Top canonical pathways for 55 OE genes by *eLNNpairedCov.SEM*

Name	p-value
Ribonucleotide Reductase Signaling Pathway	$5.34E-03$
Leukocyte Extravasation Signaling	$7.57E-03$
Cell Cycle: G1/S Checkpoint Regulation	$8.85E-03$
Tetrahydrofolate Salvage from 5,10- methenyltetrahydrofolate	$1.04E-02$
Role of Osteoblasts, Osteoclasts and Chondrocytes in Rheumatoid Arthritis	$1.19E-02$

### Results of the simulation study

**Fig. 3:**
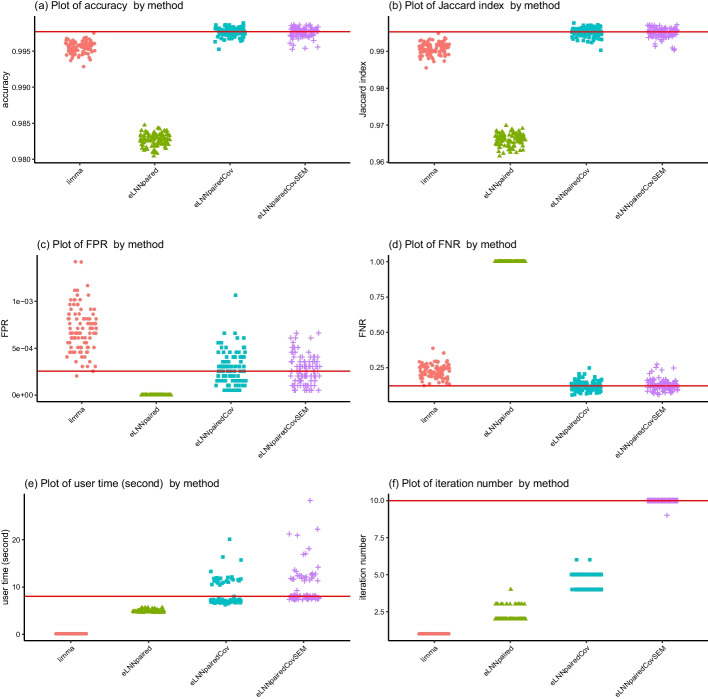
Jittered scatter plots of performance indices versus method for Set 1, Scenario 1 (number of pairs$=30$). Red solid horizontal lines indicate the median performance indices of *eLNNpairedCov.SEM*

**Fig. 4:**
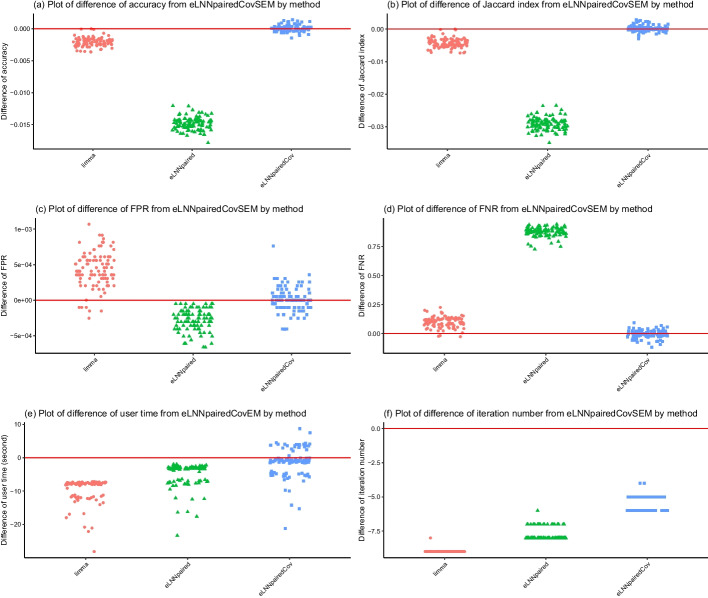
Jittered scatter plots of difference of performance indices versus method for Set 1, Scenario 1 (number of pairs$=30$). Red solid horizontal lines indicate y-axis equal to zero

**Fig. 5:**
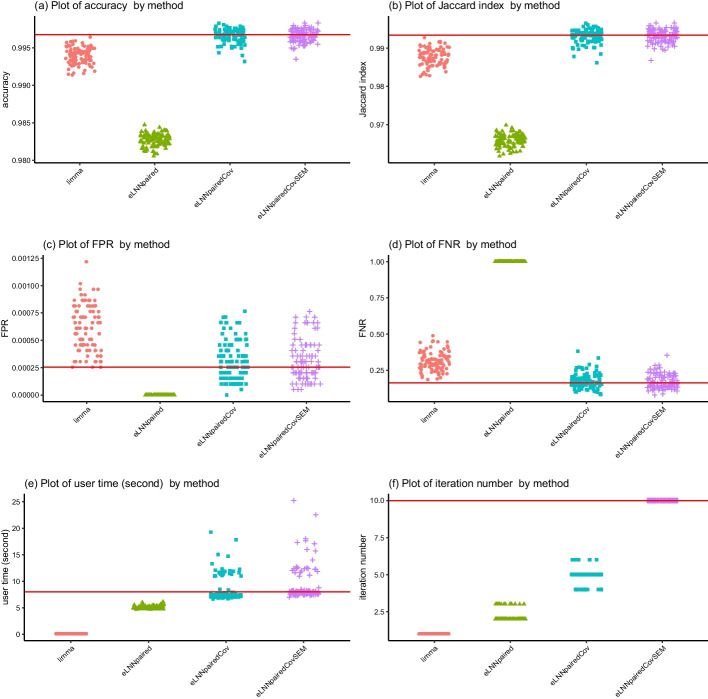
Jittered scatter plots of performance indices versus method for Set 2, Scenario 1 (number of pairs$=30$). Red solid horizontal lines indicate the median performance indices of *eLNNpairedCov.SEM*

**Fig. 6:**
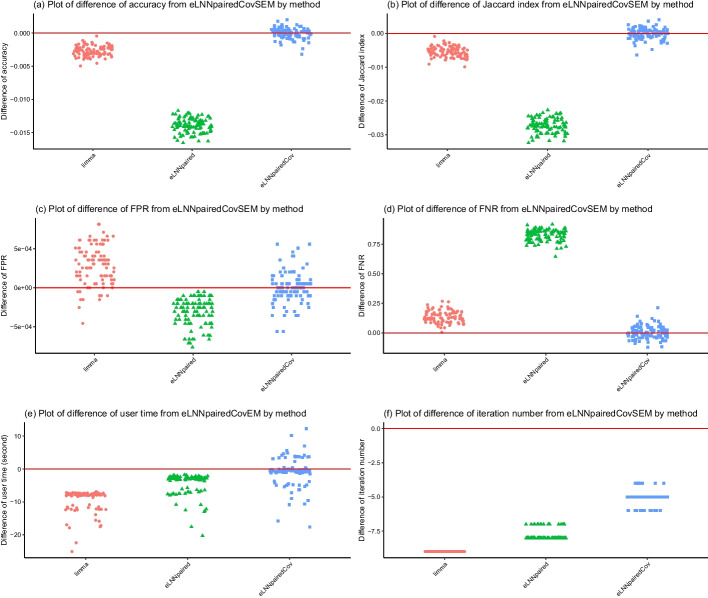
Jittered scatter plots of difference of performance indices versus method for Set 2, Scenario 1 (number of pairs$=30$). Red solid horizontal lines indicate y-axis equal to zero

For Scenario 1 ($n=30$), the jittered scatter plots of the performance indices versus methods are shown in Fig. 3 (Set 1) and Fig. 5 (Set 2) and the jittered scatter plots of the difference of the performance indices versus methods are shown in Fig. 4 (Set 1) and Figure 6 (Set 2).

The differences of performance indices are between *eLNNpairedCov.SEM* and the other three methods (*limma*, *eLNNpaired* and *eLNNpairedCov*). A positive difference indicates that the performance indices of the other method is larger than that of *eLNNpairedCov.SEM*. A negative difference indicates that the performance indices of the other method is smaller than that of *eLNNpairedCov.SEM*.

The upper panel of Figs. 3, 4, 5 and 6 show that both the *eLNNpairedCov* and *eLNNpairedCov.SEM* have higher agreement indices (Jaccard and accuracy) than *limma*, which in turn have higher agreement indices than *eLNNpaired*.

The middle panel of Figures 3-6 show that the proposed *eLNNpairedCov* and *eLNNpairedCov.SEM* methods have similar performance, They have lower FPR than *limma*, while *eLNNpaired* has an exceedingly low FPR (close to 0). The middle panel also show that *eLNNpairedCov*, *eLNNpairedCov.SEM* have smaller FNR than *limma*, while *eLNNpaired* has an exceedingly high FNR (close to 1). The extreme values in FPR and FNR of *eLNNpaired* can be attributed to the fact that it did not detect any differentially expressed genes in this case.

Additionally, Figs. 3, 4, 5 and 6 also show that compared with the performances of these methods in Set 1 (${\eta _{10}}_1$ $={\eta }_{20} =$ 0.18), those in Set 2 (${\eta _{10}}_1$ $={\eta }_{20} =$ 0.08) have lower agreement indices and higher error rates except for *eLNNpaired*, which fails to detect any differentially expressed genes in both Set 1 and Set 2.

The bottom panel of Figs. 3 and 5 show that *limma* runs very fast, while *eLNNpaired*, *eLNNpairedCov* and *eLNNpairedCov.SEM* run in reasonable time (i.e., less than 30 s per dataset that has $G=20,000$ genes and $n=30$ subjects). On average *eLNNpairedCov* and *eLNNpairedCov.SEM* spend a little more time than *eLNNpaired*. The bottom panel of Fig. 3 and 5 also show that *eLNNpaired* uses less than 5 EM iterations, while *eLNNpairedCov* and *eLNNpairedCov.SEM* tend to use more EM iterations. In particular, *eLNNpairedCov.SEM* uses 10 EM iterations, which is the maximum number of iterations we set to save computing time. Note that the EM iteration number for *limma* is set to be one, which does not use EM algorithm to obtain parameter estimates.

The simulation results for Scenario 2 ($n=100$) are shown in Figures A5-A8 [see Additional file 1], which have similar patterns to those for Scenario 1 ($n=30$), except that both *eLNNpairedCov* and *eLNNpairedCov.SEM* have smaller FPR which are close to 0. Note that *eLNNpairedCov*,*eLNNpairedCov.SEM* and *limma* have small FNR (close to 0), while *eLNNpaired* still has huge FNR (close to 1).

## Discussion and conclusion

In this article, we proposed a novel model-based clustering approach to detect differential expressed transcripts between samples before treatment and samples after treatment, with the capacity to adjust for potential confounding factors. This is novel in that to the best of our knowledge, all existing model-based gene clustering methods do not yet have the capacity to adjust for covariates.

The proposed approach is different from transcript-wise test followed by multiplicity adjustment in that it does not involve hypothesis testing. Hence, no multiplicity adjustment is needed. The simulation study showed that if the difference of gene expression between samples before treatment and samples after treatment follows the mixture of hierarchical models in Subsection “*A mixture of hierarchical models*”, then the proposed method can outperform *limma*, which is a fast and powerful transcript-wise test method. The real data analysis also showed the proposed method *eLNNpairedCov* can detect more differentially expressed gene transcripts, which include the transcripts detected by *limma*.

Although we classify genes to three distinct clusters, the transitions between these clusters could be smooth. This would be reflected by a gene’s posterior probability that might be large in two of three clusters, e.g., 0.49 for cluster 1, 0.01 for cluster 2, and 0.5 for cluster 3. On the other hand, expression changes could be split up into more than 3 clusters, e.g., groups behaving differently. In this article, we are only interested in identifying three clusters of genes: over-expressed in condition 1, under-expressed in condition 1, and non-differentially expressed.

There are other model-based clustering methods in literature, such as [40]. However, they were not designed to detect differentially expressed genes. For example, we can set the number *K* of clusters as 3 for their model. However, there is no constraints that the intercepts for the three clusters have to be positive, negative, and zero. That is, the three clusters identified might not correspond to over-expressed, under-expressed, and non-differentially expressed genes.

It is well-known in literature that EM algorithm might stuck at local optimal solution. In this article, we used EM with SA-modification to help escape from local optimal solutions. In future, we plan to try the hybrid algorithm of the DPSO (Discrete Particle Swarm Optimization) and the EM approach to improve the global search performance [41].

In our models, the three gene groups allow to have different coefficients of covariates. In future, we could test if these coefficients are same or not. If no significant difference, we could use a model assuming equal coefficients.

RNAseq and single-cell RNAseq data are cutting-edge tools to investigate molecular mechanisms of complex human diseases. However, it is quite challenging to analyze these count data with inflated zero counts. In future, we will evaluate if eLNNpairedCov can be used to analyze single-cell RNAseq data by first transforming counts to continuous scale (e.g., via VOOM [12] or countTransformers [13]) and then to apply eLNNpairedCov to the transformed data.

We implemented the proposed methods to an R package *eLNNpairedCov*, which will be freely available to researchers.

### Supplementary Information

**Additional file 1.** Supplementary Document.

**Additional file 2:** Table S1.Gene list of 6 UE transcripts detected by limma.

**Additional file 3:** Table S2.Gene list of 55 OE transcripts detected by eLNNpairedCov.

**Additional file 4:** Table S3.Gene list of 59 OE transcripts detected by eLNNpairedCov.SEM.

**Additional file 5:** Table S4.Gene list of 355 UE transcripts detected by eLNNpairedCov.

**Additional file 6:** Table S5.Gene list of 352 UE transcripts detected by eLNNpairedCov.SEM.

## Data Availability

The real dataset analyzed during the current study are available in the Gene Expression Omnibus (GEO) repository, [https://www.ncbi.nlm.nih.gov/geo/query/acc.cgi?acc=GSE24742].
