# Supplementary material for: A model-based clustering via mixture of hierarchical models with covariate adjustment for detecting differentially expressed genes from paired design
Source: BMC Bioinformatics. 2023 Nov 8;24:423. doi: 10.1186/s12859-023-05556-x (PMC10633962; doi:10.1186/s12859-023-05556-x)
Supplement: Supplementary file 1 — Additional file 1. Supplementary Document. [file 12859_2023_5556_MOESM1_ESM.pdf]

Supplementary Document for "A Model-Based Clustering via Mixture of Hierarchical Models with Covariate Adjustment for Detecting Differentially Expressed Genes from Paired Design"

Yixin Zhang[1] and Wei Liu[2]*and Weiliang Qiu[3]
[1]: University of Science and Technology of China, School of Mathematical Science, Hefei, Anhui 230000, China
[2]: York University, Department of Mathematics and Statistics, Toronto, ON M3J 1P3, Canada
[3]: Sanofi, Department of Biostatistics and Programming, Cambridge, MA 02141, USA

*liuwei@yorku.ca

A Histograms of gene expression for GSE24742

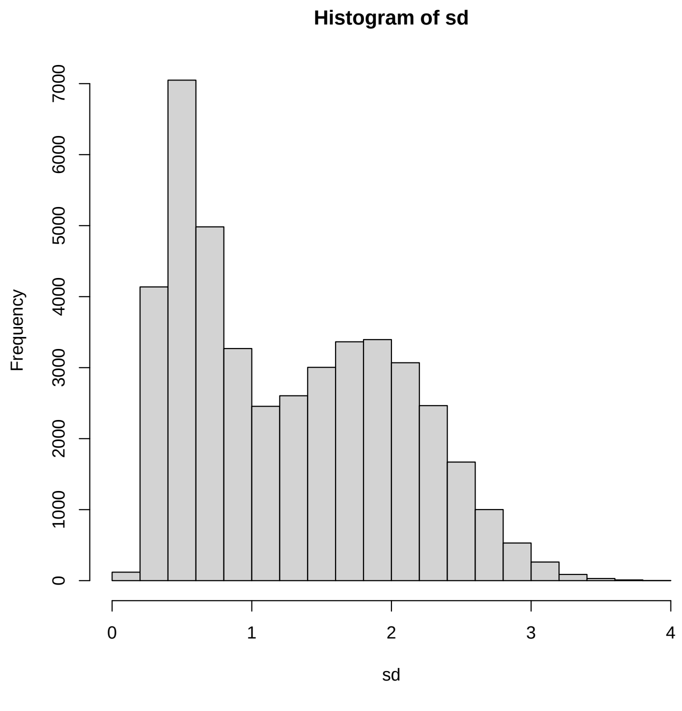

Figure A1: Histogram of the estimated standard deviations of log2 within-subject differences of gene expression for 43,506 gene probes in GSE24742.

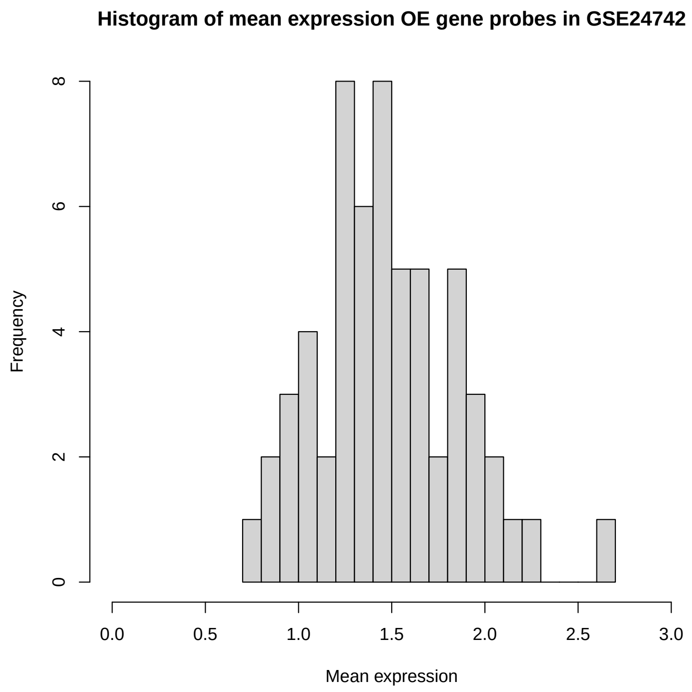

Figure A2: Histogram of the mean log2 within-subject differences of gene expression for 59 UE gene probes detected by *eLNNpairedCov.SEM* in GSE24742.

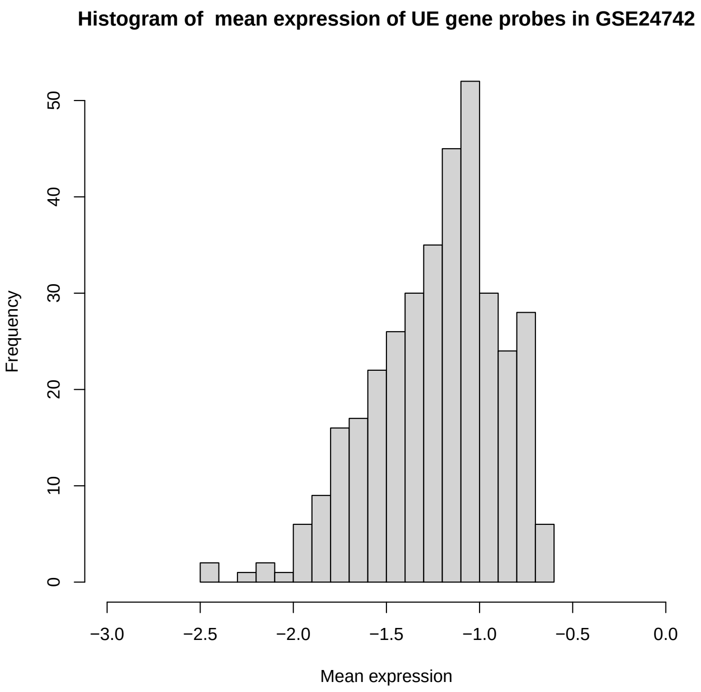

Figure A3: Histogram of the mean log2 within-subject differences of gene expression for 352 UE gene probes detected by *eLNNpairedCov.SEM* in GSE24742.

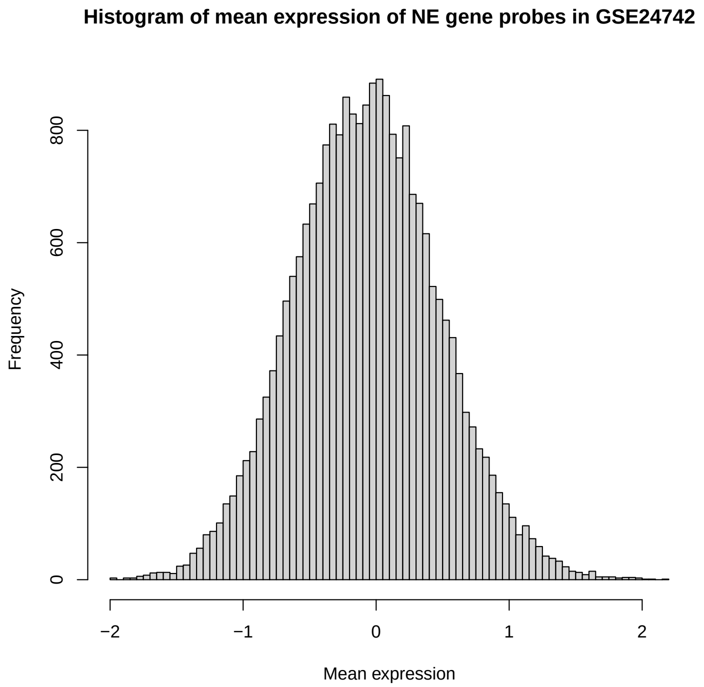

Figure A4: Histogram of the mean log2 within-subject differences of gene expression for 23537 NE gene probes detected by *eLNNpairedCov.SEM* in GSE24742.

B Additional results

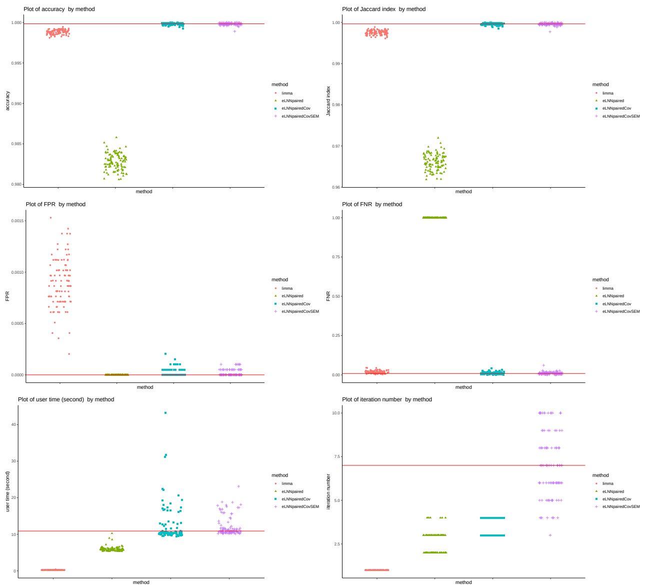

Figure A5: Jittered scatter plots of performance indices versus method for Set1,Scenario2 (number of pairs= 100). Red solid horizontal lines indicate the median performance indices of *eLNNpairedCov.SEM*.

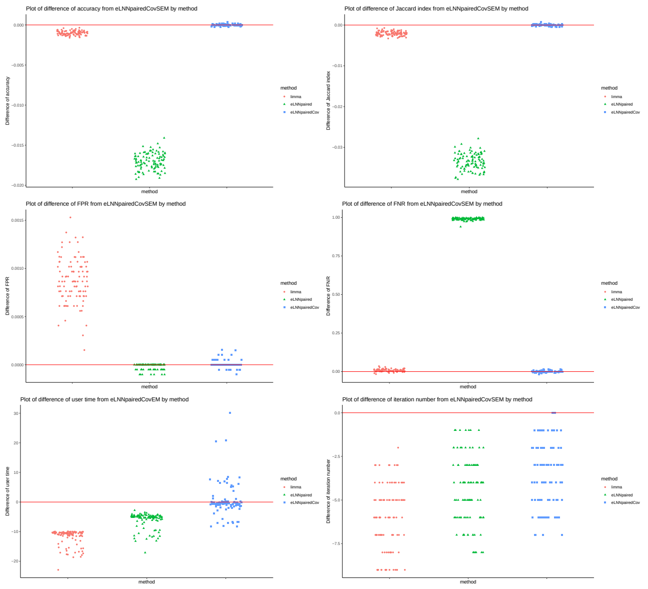

Figure A6: Jittered scatter plots of difference of performance indices versus method for Set1, Scenario2 (number of pairs= 100). Red solid horizontal lines indicate y-axis equal to zero.

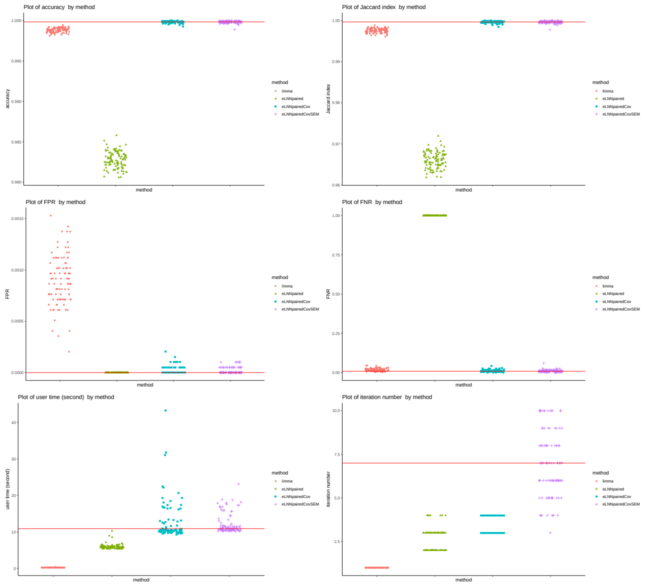

Figure A7: Jittered scatter plots of performance indices versus method for Set2, Scenario2 (number of pairs= 100). Red solid horizontal lines indicate the median performance indices of *eLNNpairedCov.SEM*.

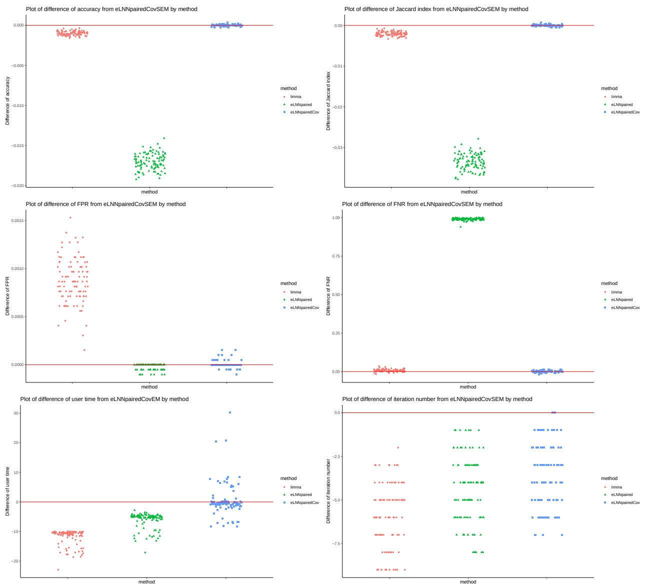

Figure A8: Jittered scatter plots of difference of performance indices versus method for Set2, Scenario2 (number of pairs= 100). Red solid horizontal lines indicate y-axis equal to zero.

C Marginal density functions

The marginal density function of $\mathbf{d}_g$is :

$$f(\mathbf{d}_g|\boldsymbol{\psi}) = \pi_1 f_1(\mathbf{d}_g|\boldsymbol{\psi}) + \pi_2 f_2(\mathbf{d_g}|\boldsymbol{\psi}) + \pi_3 f_3(\mathbf{d}_g|\boldsymbol{\psi})$$

where $f_1(\mathbf{d}_g|\boldsymbol{\psi})$, $f_2(\mathbf{d}_g|\boldsymbol{\psi})$, $f_3(\mathbf{d}_g|\boldsymbol{\psi})$ are the marginal densities of the 3 clusters, and $\boldsymbol{\pi} = (\pi_1, \pi_2, \pi_3)$ is the cluster proportion.

Then we have the marginal distribution for each cluster:

$$\begin{aligned}
f_1(\boldsymbol{d}_g|\boldsymbol{\psi}) &= \int_{-\infty}^{+\infty}\int_0^{\infty} N(\boldsymbol{d}_g;\boldsymbol{\mu}_g,\tau_g^{-1}\boldsymbol{I}_n)N(\boldsymbol{\mu}_g;\exp(\boldsymbol{W}^T\boldsymbol{\eta}_1),k_1\tau_g^{-1}\boldsymbol{I}_n)\Gamma(\alpha_1,\beta_1)d\boldsymbol{\mu}_g d\tau_g \\
&= \frac{\beta_1^{\alpha_1}}{|2\pi|^{\frac{n}{2}}\Gamma(\alpha_1)}(k_1+1)^{-\dfrac{n}{2}}\int_0^{\infty}\tau_g^{\frac{n}{2}+\alpha_1-1}e^{-\tau_g(\beta_1-\boldsymbol{M}_1)} \\
&= \frac{\beta_1^{\alpha_1}\Gamma(\frac{n}{2}+\alpha_1)}{|2\pi|^{\frac{n}{2}}\Gamma(\alpha_1)}(k_1+1)^{-\frac{n}{2}}(\beta_1-\boldsymbol{M}_1)^{-(\frac{n}{2}+\alpha_1)},
\end{aligned}$$

$$\begin{aligned}
f_2(\boldsymbol{d}_g|\boldsymbol{\psi}) &= \int_{-\infty}^{+\infty}\int_0^{\infty} N(\boldsymbol{d}_g;\boldsymbol{\mu}_g,\tau_g^{-1}\boldsymbol{I}_n)N(\boldsymbol{\mu}_g;-\exp(\boldsymbol{W}^T\boldsymbol{\eta}_2),k_2\tau_g^{-1}\boldsymbol{I}_n)\Gamma(\alpha_2,\beta_2)d\boldsymbol{\mu}_g d\tau_g \\
&= \frac{\beta_2^{\alpha_2}\Gamma(\frac{n}{2}+\alpha_2)}{|2\pi|^{\frac{n}{2}}\Gamma(\alpha_2)}(k_2+1)^{-\frac{n}{2}}(\beta_2-\boldsymbol{M}_2)^{-(\frac{n}{2}+\alpha_2)},
\end{aligned}$$

where $\boldsymbol{W}^T$ is the $n \times (p+1)$ design matrix.

$$
\begin{aligned}
f_3(\mathbf{d}_g|\boldsymbol{\psi}) =& \int_0^{\infty}\int_{-\infty}^{\infty} N(\boldsymbol{d}_g;\boldsymbol{U}^T\boldsymbol{\theta}_g,\tau_g\boldsymbol{I}_n)N(\boldsymbol{\theta}_g;\boldsymbol{\eta}_3,k_3\tau_g^{-1}\boldsymbol{I}_p G(\tau_g;\alpha_3,\beta_3)d\boldsymbol{\theta}_g d\tau_g \\
=& \frac{\beta_3^{\alpha_3}\Gamma(\frac{n}{2}+\alpha_3)}{\Gamma(\alpha_3)(2\pi)^{\frac{n}{2}}}|\boldsymbol{I}_p + k_3\boldsymbol{U}\boldsymbol{U}^T|^{-\frac{1}{2}} \\
& \left[\beta_3 + \frac{1}{2}\boldsymbol{d}_g^T\boldsymbol{d}_g + \frac{1}{2k_3}\boldsymbol{\eta}_3^T\boldsymbol{\eta}_3 \right. \\
& \left. -\frac{1}{2k_3}(k_3\boldsymbol{U}\boldsymbol{d}_g+\boldsymbol{\eta}_3)^T\left(\boldsymbol{I}_p+k_3\boldsymbol{U}\boldsymbol{U}^T\right)^{-1}(k_3\boldsymbol{U}\boldsymbol{d}_g+\boldsymbol{\eta}_3)\right]^{-(\frac{n}{2}+\alpha_3)},
\end{aligned}
$$

where $\boldsymbol{U}^T$ is the $n \times p$ design matrix without intercept column.

Here

$$
\begin{aligned}
\boldsymbol{M}_1 =& \frac{1}{2}\left(\boldsymbol{d}_g^T\boldsymbol{I}_n + (e^{\boldsymbol{W}^T\boldsymbol{\eta}_1})^T\frac{1}{k_1}\boldsymbol{I}_n\right)(\boldsymbol{I}_n+\frac{1}{k_1}\boldsymbol{I}_n)^{-1}\left(\boldsymbol{I}_n\boldsymbol{d}_g+\frac{1}{k_1}\boldsymbol{I}_n e^{\boldsymbol{W}^T\boldsymbol{\eta}_1}\right) \\
& -\frac{1}{2}\left(\boldsymbol{d}_g^T\boldsymbol{I}_n\boldsymbol{d}_g + (e^{\boldsymbol{W}^T\boldsymbol{\eta}_1})^T\frac{1}{k_1}\boldsymbol{I}_n e^{\boldsymbol{W}^T\boldsymbol{\eta}_1}\right) \\
=& -\frac{1}{2}\frac{1}{k_1+1}\boldsymbol{d}_g^T\boldsymbol{d}_g + \frac{1}{k_1+1}\boldsymbol{d}_g^T e^{\boldsymbol{W}^T\boldsymbol{\eta}_1} - \frac{1}{2}\frac{1}{k_1+1}\left(e^{\boldsymbol{W}^T\boldsymbol{\eta}_1}\right)^T e^{W^T\boldsymbol{\eta}_1}
\end{aligned}
$$

$$
\begin{aligned}
\boldsymbol{M}_2 =& \frac{1}{2}\left(\boldsymbol{d}_g^T\boldsymbol{I}_n - (e^{\boldsymbol{W}^T\boldsymbol{\eta}_2})^T\frac{1}{k_2}\boldsymbol{I}_n\right)(\boldsymbol{I}_n+\frac{1}{k_2}\boldsymbol{I}_n)^{-1}\left(\boldsymbol{I}_n\boldsymbol{d}_g-\frac{1}{k_2}\boldsymbol{I}_n e^{\boldsymbol{W}^T\boldsymbol{\eta}_2}\right) \\
& -\frac{1}{2}\left(\boldsymbol{d}_g^T\boldsymbol{I}_n\boldsymbol{d}_g + (e^{\boldsymbol{W}^T\boldsymbol{\eta}_2})^T\frac{1}{k_2}\boldsymbol{I}_n e^{\boldsymbol{W}^T\boldsymbol{\eta}_2}\right) \\
=& -\frac{1}{2}\frac{1}{k_2+1}\boldsymbol{d}_g^T\boldsymbol{d}_g - \frac{1}{k_2+1}\boldsymbol{d}_g^T e^{\boldsymbol{W}^T\boldsymbol{\eta}_2} - \frac{1}{2}\frac{1}{k_2+1}\left(e^{\boldsymbol{W}^T\boldsymbol{\eta}_2}\right)^T e^{\boldsymbol{W}^T\boldsymbol{\eta}_2}
\end{aligned}
$$

Note that each marginal density is the density of a multivariate t distribution with special structures of mean vector and covariance matrix.

D Log marginal densities

$$
\begin{aligned}
\log\left[f_1\left(\boldsymbol{d}_g|\boldsymbol{\psi}\right)\right] =& \alpha_1\log\left(\beta_1\right) + \log\left[\Gamma\left(\frac{n}{2}+\alpha_1\right)\right] - \frac{n}{2}\log(k_1+1) - \left(\frac{n}{2}+\alpha_1\right)\log\left(\beta_1-\boldsymbol{M}_1\right) \\
& -\frac{n}{2}\log\left(2\pi\right) - \log\left[\Gamma\left(\alpha_1\right)\right]
\end{aligned}
$$

$$
\begin{aligned}
\log\left[f_2\left(\boldsymbol{d}_g|\boldsymbol{\psi}\right)\right] =& \alpha_2\log\left(\beta_2\right) + \log\left[\Gamma\left(\frac{n}{2}+\alpha_2\right)\right] - \frac{n}{2}\log(k_2+1) - \left(\frac{n}{2}+\alpha_2\right)\log\left(\beta_2-\boldsymbol{M}_2\right) \\
& -\frac{n}{2}\log\left(2\pi\right) - \log\left[\Gamma\left(\alpha_2\right)\right]
\end{aligned}
$$

$$
\begin{aligned}
\log\left[f_3\left(\boldsymbol{d}_g|\boldsymbol{\psi}\right)\right] =& \alpha_3 \log\left(\beta_3\right) + \log\left[\Gamma\left(\frac{n}{2}+\alpha_3\right)\right] - \frac{n}{2}\log\left(2\pi\right) - \log\left[\Gamma\left(\alpha_3\right)\right] \\
& - \frac{1}{2}\log|\boldsymbol{I}_p + k_3\boldsymbol{U}\boldsymbol{U}^T| \\
& - \left(\frac{n}{2}+\alpha_3\right)\log(\beta_3+\boldsymbol{M}_3)
\end{aligned}
$$

Here

$$
\boldsymbol{M}_3 = \frac{1}{2}\boldsymbol{d}_g^T\boldsymbol{d}_g + \frac{1}{2k_3}\boldsymbol{\eta}_3^T\boldsymbol{\eta}_3 - \frac{1}{2k_3}(k_3\boldsymbol{U}\boldsymbol{d}_g+\boldsymbol{\eta}_3)^T\left(\boldsymbol{I}_p + k_3\boldsymbol{U}\boldsymbol{U}^T\right)^{-1}(k_3\boldsymbol{U}\boldsymbol{d}_g+\boldsymbol{\eta}_3)
$$

E First Derivatives

For π_1, π_2, π_3, we have:

$$
\begin{aligned}
\frac{\partial Q^{(t)}}{\partial \pi_1} &= \sum_g \left(\frac{\zeta_{g1}^{(t)}}{\pi_1} - \frac{\zeta_{g3}^{(t)}}{1-\pi_1-\pi_2}\right) + \frac{b_1-1}{\pi_1} - \frac{b_3-1}{1-\pi_1-\pi_2} \\
\frac{\partial Q^{(t)}}{\partial \pi_2} &= \sum_g \left(\frac{\zeta_{g2}^{(t)}}{\pi_2} - \frac{\zeta_{g3}^{(t)}}{1-\pi_1-\pi_2}\right) + \frac{b_2-1}{\pi_2} - \frac{b_3-1}{1-\pi_1-\pi_2}
\end{aligned}
$$

Since $\pi_1+\pi_2+\pi_3 = 1$, $\sum_g(\zeta_{g1}^{(t)}+\zeta_{g2}^{(t)}+\zeta_{g3}^{(t)}) = G$, and letting $b_1 = b_2 = b_3 = 2$, $\partial Q^{(t)}/\partial\pi_1 = \partial Q^{(t)}/\partial\pi_2 = 0$, we have:

$$
\frac{\sum\zeta_{g1}^{(t)}+1}{\pi_1} = \frac{\sum\zeta_{g2}^{(t)}+1}{\pi_2} = \frac{\sum\zeta_{g3}^{(t)}+1}{\pi_3}
$$

$$
\pi_1^{(t+1)} = \frac{\sum\zeta_{g1}^{(t)}+1}{G+3}, \pi_2^{(t+1)} = \frac{\sum\zeta_{g2}^{(t)}+1}{G+3}, \pi_3^{(t+1)} = \frac{\sum\zeta_{g3}^{(t)}+1}{G+3}
$$

E.1 For OE genes

For α_1, β_1, k_1 and $\boldsymbol{\eta}_1$, we have

$$
\begin{aligned}
\frac{\partial Q^{(t)}}{\partial \alpha_1} &= \sum_g \zeta_{g1}^{(t)}\frac{\partial \log f_1}{\partial \alpha_1} \\
\frac{\partial Q^{(t)}}{\partial \beta_1} &= \sum_g \zeta_{g1}^{(t)}\frac{\partial \log f_1}{\partial \beta_1} \\
\frac{\partial Q^{(t)}}{\partial k_1} &= \sum_g \zeta_{g1}^{(t)}\frac{\partial \log f_1}{\partial k_1} \\
\frac{\partial Q^{(t)}}{\partial \boldsymbol{\eta}_1} &= \sum_g \zeta_{g1}^{(t)}\frac{\partial \log f_1}{\partial \boldsymbol{\eta}_1}
\end{aligned}
$$

Now, we would like to take partial derivatives of $\log f_1$ with respect to α_1, β_1, k_1 and $\boldsymbol{\eta}_1$. And we have:

$$\begin{aligned}\log\left[f_1\left(\boldsymbol{d}_g|\boldsymbol{\psi}\right)\right] =& \alpha_1 \log\left(\beta_1\right) + \log\left[\Gamma\left(\frac{n}{2}+\alpha_1\right)\right] - \frac{n}{2}\log(k_1+1) - \left(\frac{n}{2}+\alpha_1\right)\log\left(\beta_1 - \boldsymbol{M}_1\right) \\ & - \frac{n}{2}\log\left(2\pi\right) - \log\left[\Gamma\left(\alpha_1\right)\right]\end{aligned}$$

$$\frac{\partial \log\left(f_1\right)}{\partial \alpha_1} = \log\left(\beta_1\right) + digamma\left(\frac{n}{2}+\alpha_1\right) - \log\left(\beta_1 - \boldsymbol{M}_1\right) - digamma\left(\alpha_1\right)$$

$$digamma(x) = d\log(\Gamma(x))/dx$$

$$\frac{\partial \log\left(f_1\right)}{\partial \beta_1} = \frac{\alpha_1}{\beta_1} - \frac{\left(\frac{n}{2}+\alpha_1\right)}{\left(\beta_1 - \boldsymbol{M}_1\right)}$$

$$\frac{\partial \log\left(f_1\right)}{\partial \boldsymbol{\eta}_1} = \frac{\left(\frac{n}{2}+\alpha_1\right)}{\left(\beta_1 - \boldsymbol{M}_1\right)}\frac{\partial \boldsymbol{M}_1}{\partial \boldsymbol{\eta}_1}$$

Denote

$$\boldsymbol{\xi}_1 = \exp\left(\boldsymbol{W}^T\boldsymbol{\eta}_1\right) = \begin{pmatrix} \exp\left(\boldsymbol{W}_1^T\boldsymbol{\eta}_1\right) \\ \vdots \\ \exp\left(\boldsymbol{W}_n^T\boldsymbol{\eta}_1\right)\end{pmatrix},$$

where

$$\boldsymbol{W}_l = \begin{pmatrix} w_{l0} \\ w_{l1} \\ \vdots \\ w_{lp}\end{pmatrix}, \quad l = 1, \ldots, n.$$

Then we can rewrite $\boldsymbol{M}_1$ as

$$\boldsymbol{M}_1 = -\frac{1}{2}\frac{1}{k_1+1}\boldsymbol{d}_g^T\boldsymbol{d}_g + \frac{1}{k_1+1}\boldsymbol{d}_g^T\boldsymbol{\xi}_1 - \frac{1}{2}\frac{1}{k_1+1}\boldsymbol{\xi}_1^T\boldsymbol{\xi}_1$$

$$\frac{\partial \boldsymbol{M}_1}{\partial \boldsymbol{\eta}_1} = \frac{1}{k_1+1}\boldsymbol{W}diag(\boldsymbol{d}_g)\boldsymbol{\xi}_1 - \frac{1}{k_1+1}\boldsymbol{X}diag(\boldsymbol{\xi}_1)\boldsymbol{\xi}_1$$

As for k_1, we have

$$\frac{\partial \log f_1}{\partial k_1} = -\frac{n}{2}\frac{1}{k_1+1} + \frac{\frac{n}{2}+\alpha_1}{\beta_1 - \boldsymbol{M}_1}\frac{\partial \boldsymbol{M}_1}{\partial k_1}$$

$$\frac{\partial \boldsymbol{M}_1}{\partial k_1} = \frac{1}{2}\frac{1}{(k_1+1)^2}\boldsymbol{d}_g^T\boldsymbol{d}_g - \frac{1}{(k_1+1)^2}\boldsymbol{d}_g^T\boldsymbol{\xi}_1 + \frac{1}{2}\frac{1}{(k_1+1)^2}\boldsymbol{\xi}_1^T\boldsymbol{\xi}_1$$

E.2 For UE gene probes

For α_2, β_2, k_2 and $\boldsymbol{\eta}_2$, we have

$$\frac{\partial Q^{(t)}}{\partial \alpha_2} = \sum_g \zeta_{g2}^{(t)} \frac{\partial \log f_2}{\partial \alpha_2}$$

$$\frac{\partial Q^{(t)}}{\partial \beta_2} = \sum_g \zeta_{g2}^{(t)} \frac{\partial \log f_2}{\partial \beta_2}$$

$$\frac{\partial Q^{(t)}}{\partial k_2} = \sum_g \zeta_{g2}^{(t)} \frac{\partial \log f_2}{\partial k_2}$$

$$\frac{\partial Q^{(t)}}{\partial \boldsymbol{\eta}_2} = \sum_g \zeta_{g2}^{(t)} \frac{\partial \log f_2}{\partial \boldsymbol{\eta}_2}$$

Take partial derivatives of $\log f_2$ with respect to α_2, β_2, k_2 and $\boldsymbol{\eta}_2$. And we have:

$$\begin{aligned}\log\left[f_2\left(\boldsymbol{d}_g|\boldsymbol{\psi}\right)\right] =& \alpha_2 \log\left(\beta_2\right) + \log\left[\Gamma\left(\frac{n}{2}+\alpha_2\right)\right] - \frac{n}{2}\log(k_2+1) - \left(\frac{n}{2}+\alpha_2\right)\log\left(\beta_2 - \boldsymbol{M}_2\right) \\ & - \frac{n}{2}\log\left(2\pi\right) - \log\left[\Gamma\left(\alpha_2\right)\right]\end{aligned}$$

$$\frac{\partial \log\left(f_2\right)}{\partial \alpha_2} = \log\left(\beta_2\right) + digamma\left(\frac{n}{2}+\alpha_2\right) - \log\left(\beta_2 - \boldsymbol{M}_2\right) - digamma\left(\alpha_2\right)$$

$$\frac{\partial \log\left(f_2\right)}{\partial \beta_2} = \frac{\alpha_2}{\beta_2} - \frac{\left(\frac{n}{2}+\alpha_2\right)}{\left(\beta_2 - \boldsymbol{M}_2\right)}$$

$$\frac{\partial \log\left(f_2\right)}{\partial \boldsymbol{\eta}_2} = \frac{\left(\frac{n}{2}+\alpha_2\right)}{\left(\beta_2 - \boldsymbol{M}_2\right)} \frac{\partial \boldsymbol{M}_2}{\partial \boldsymbol{\eta}_2}$$

Denote

$$\boldsymbol{\xi}_2 = \exp\left(\boldsymbol{W}^T\boldsymbol{\eta}_2\right) = \begin{pmatrix} \exp\left(\boldsymbol{W}_1^T\boldsymbol{\eta}_2\right) \\ \vdots \\ \exp\left(\boldsymbol{W}_n^T\boldsymbol{\eta}_2\right)\end{pmatrix},$$

where

$$\boldsymbol{W}_l = \begin{pmatrix} 1 \\ w_{l1} \\ \vdots \\ w_{lp}\end{pmatrix}, \quad l = 1, \ldots, n.$$

Then we can rewrite $\boldsymbol{M}_2$ as

$$\boldsymbol{M}_2 = -\frac{1}{2}\frac{1}{k_2+1}\boldsymbol{d}_g^T\boldsymbol{d}_g - \frac{1}{k_2+1}\boldsymbol{d}_g^T\boldsymbol{\xi}_2 - \frac{1}{2}\frac{1}{k_2+1}\boldsymbol{\xi}_2^T\boldsymbol{\xi}_2$$

$$\frac{\partial \boldsymbol{M}_2}{\partial \boldsymbol{\eta}_2} = -\frac{1}{k_2+1}\boldsymbol{W} diag(\boldsymbol{d}_g)\boldsymbol{\xi}_2 - \frac{1}{k_2+1}\boldsymbol{W} diag(\boldsymbol{\xi}_2)\boldsymbol{\xi}_2$$

For k_2, we have

$$\frac{\partial \log f_2}{\partial k_2} = -\frac{n}{2}\frac{1}{k_2+1} + \frac{\frac{n}{2}+\alpha_2}{\beta_2 - \boldsymbol{M}_2}\frac{\partial \boldsymbol{M}_2}{\partial k_2}$$

$$\frac{\partial \boldsymbol{M}_2}{\partial k_2} = \frac{1}{2}\frac{1}{(k_2+1)^2}\boldsymbol{d}_g^T\boldsymbol{d}_g + \frac{1}{(k_2+1)^2}\boldsymbol{d}_g^T\boldsymbol{\xi}_2 + \frac{1}{2}\frac{1}{(k_2+1)^2}\boldsymbol{\xi}_2^T\boldsymbol{\xi}_2$$

E.3 For NE gene probes

For α_3, β_3, we have

$$\begin{aligned}
\frac{\partial Q^{(t)}}{\partial \alpha_3} &= \sum_g \zeta_{g3}^{(t)}\frac{\partial \log f_3}{\partial \alpha_3} \\
\frac{\partial Q^{(t)}}{\partial \beta_3} &= \sum_g \zeta_{g3}^{(t)}\frac{\partial \log f_3}{\partial \beta_3} \\
\log\left[f_3\left(\boldsymbol{d}_g|\boldsymbol{\psi}\right)\right] &= \alpha_3\log\left(\beta_3\right) + \log\left[\Gamma\left(\frac{n}{2}+\alpha_3\right)\right] - \left(\frac{n}{2}+\alpha_3\right)\log\left(\beta_3+\boldsymbol{M}_3\right) \\
&\quad - \frac{n}{2}\log\left(2\pi\right) - \log\left[\Gamma\left(\alpha_3\right)\right]
\end{aligned}$$

Here

$$\boldsymbol{M}_3 = \frac{1}{2}\boldsymbol{d}_g^T\boldsymbol{d}_g + \frac{1}{2k_3}\boldsymbol{\eta}_3^T\boldsymbol{\eta}_3 - \frac{1}{2k_3}(k_3\boldsymbol{U}\boldsymbol{d}_g+\boldsymbol{\eta}_3)^T\left(\boldsymbol{I}_p + k_3\boldsymbol{U}\boldsymbol{U}^T\right)^{-1}(k_3\boldsymbol{U}\boldsymbol{d}_g+\boldsymbol{\eta}_3)$$

Therefore, take the derivatives of $\log f_3$, we have

$$\frac{\partial \log f_3}{\partial \alpha_3} = \log\beta_3 + digamma\left(\frac{n}{2}+\alpha_3\right) - \log\left(\beta_3+\boldsymbol{M}_3\right) - digamma(\alpha_3)$$

$$\begin{aligned}
\frac{\partial \log f_3}{\partial \beta_3} &= \frac{\alpha_3}{\beta_3} - \left(\frac{n}{2}+\alpha_3\right)\frac{1}{\beta_3+\boldsymbol{M}_3} \\
\frac{\partial \log\left(f_3\right)}{\partial \boldsymbol{\eta}_3} &= \frac{\left(\frac{n}{2}+\alpha_3\right)}{\left(\beta_3+\boldsymbol{M}_3\right)}\frac{\partial \boldsymbol{M}_3}{\partial \boldsymbol{\eta}_3}
\end{aligned}$$

For $\frac{\partial \boldsymbol{M}_3}{\partial \boldsymbol{\eta}_3}$, we consider the following two components,

$$\frac{\partial \boldsymbol{\eta}_3^T \boldsymbol{\eta}_3}{\partial \boldsymbol{\eta}_3} = 2\boldsymbol{\eta}_3$$

And we have (Matrix Cookbook [1], equation(85)), given $\mathbf{V}$ is symmetric,

$$\frac{\partial}{\partial \mathbf{s}}(\mathbf{x}-\mathbf{s})^{\mathbf{T}}\mathbf{V}(\mathbf{x}-\mathbf{s}) = 2\mathbf{V}(\mathbf{x}-\mathbf{s})$$

Then,

$$\frac{\partial}{\partial \boldsymbol{\eta}_3}(k_3\boldsymbol{U}\boldsymbol{d}_g + \boldsymbol{\eta}_3)^T(\boldsymbol{I}_p + k_3\boldsymbol{U}\boldsymbol{U}^T)^{-1}(k_3\boldsymbol{U}\boldsymbol{d}_g + \boldsymbol{\eta}_3) = 2(\boldsymbol{I}_p + k_3\boldsymbol{U}\boldsymbol{U}^T)^{-1}(k_3\boldsymbol{U}\boldsymbol{d}_g + \boldsymbol{\eta}_3)$$

$$\frac{\partial \boldsymbol{M}_3}{\partial \boldsymbol{\eta}_3} = \frac{1}{k_3}\boldsymbol{\eta}_3 - \frac{1}{k_3}(\boldsymbol{I}_p + k_3\boldsymbol{U}\boldsymbol{U}^T)^{-1}(k_3\boldsymbol{U}\boldsymbol{d}_g + \boldsymbol{\eta}_3)$$

For k_3, we have

$$\frac{\partial \log(f_3)}{\partial k_3} = \frac{\left(\frac{n}{2} + \alpha_3\right)}{(\beta_3 + \boldsymbol{M}_3)}\frac{\partial \boldsymbol{M}_3}{\partial k_3}$$

$$\begin{aligned}
&\mathrm{d}\left[(k_3\boldsymbol{U}\boldsymbol{d}_g + \boldsymbol{\eta}_3)^T(\boldsymbol{I}_p + k_3\boldsymbol{U}\boldsymbol{U}^T)^{-1}(k_3\boldsymbol{U}\boldsymbol{d}_g + \boldsymbol{\eta}_3)\right] \\
=&(\boldsymbol{U}\boldsymbol{d}_g\mathrm{d}k_3)^T(\boldsymbol{I}_p + k_3\boldsymbol{U}\boldsymbol{U}^T)^{-1}(k_3\boldsymbol{U}\boldsymbol{d}_g + \boldsymbol{\eta}_3) \\
&+ (k_3\boldsymbol{U}\boldsymbol{d}_g + \boldsymbol{\eta}_3)^T\mathrm{d}\left[(\boldsymbol{I}_p + k_3\boldsymbol{U}\boldsymbol{U}^T)^{-1}\right](k_3\boldsymbol{U}\boldsymbol{d}_g + \boldsymbol{\eta}_3) \\
&+ (k_3\boldsymbol{U}\boldsymbol{d}_g + \boldsymbol{\eta}_3)^T(\boldsymbol{I}_p + k_3\boldsymbol{U}\boldsymbol{U}^T)^{-1}(\boldsymbol{U}\boldsymbol{d}_g\mathrm{d}k_3) \\
=&2(k_3\boldsymbol{U}\boldsymbol{d}_g + \boldsymbol{\eta}_3)^T(\boldsymbol{I}_p + k_3\boldsymbol{U}\boldsymbol{U}^T)^{-1}\boldsymbol{U}\boldsymbol{d}_g\mathrm{d}k_3 \\
&+ (k_3\boldsymbol{U}\boldsymbol{d}_g + \boldsymbol{\eta}_3)^T\left[-(\boldsymbol{I}_p + k_3\boldsymbol{U}\boldsymbol{U}^T)^{-1}(\boldsymbol{U}\boldsymbol{U}^T\mathrm{d}k_3)(\boldsymbol{I}_p + k_3\boldsymbol{U}\boldsymbol{U}^T)^{-1}\right](k_3\boldsymbol{U}\boldsymbol{d}_g + \boldsymbol{\eta}_3) \\
=&2(k_3\boldsymbol{U}\boldsymbol{d}_g + \boldsymbol{\eta}_3)^T(\boldsymbol{I}_p + k_3\boldsymbol{U}\boldsymbol{U}^T)^{-1}\boldsymbol{U}\boldsymbol{d}_g\mathrm{d}k_3 \\
&+ (k_3\boldsymbol{U}\boldsymbol{d}_g + \boldsymbol{\eta}_3)^T\left[-(\boldsymbol{I}_p + k_3\boldsymbol{U}\boldsymbol{U}^T)^{-1}\boldsymbol{U}\boldsymbol{U}^T(\boldsymbol{I}_p + k_3\boldsymbol{U}\boldsymbol{U}^T)^{-1}\right](k_3\boldsymbol{U}\boldsymbol{d}_g + \boldsymbol{\eta}_3)\mathrm{d}k_3
\end{aligned}$$

$$\begin{aligned}
&\frac{\partial}{\partial k_3}(k_3\boldsymbol{U}\boldsymbol{d}_g + \boldsymbol{\eta}_3)^T(\boldsymbol{I}_p + k_3\boldsymbol{U}\boldsymbol{U}^T)^{-1}(k_3\boldsymbol{U}\boldsymbol{d}_g + \boldsymbol{\eta}_3) \\
=&2(\boldsymbol{U}\boldsymbol{d}_g)^T(\boldsymbol{I}_p + k_3\boldsymbol{U}\boldsymbol{U}^T)^{-1}(k_3\boldsymbol{U}\boldsymbol{d}_g + \boldsymbol{\eta}_3) \\
&- (k_3\boldsymbol{U}\boldsymbol{d}_g + \boldsymbol{\eta}_3)^T(\boldsymbol{I}_p + k_3\boldsymbol{U}\boldsymbol{U}^T)^{-1}\boldsymbol{U}\boldsymbol{U}^T(\boldsymbol{I}_p + k_3\boldsymbol{U}\boldsymbol{U}^T)^{-1}(k_3\boldsymbol{U}\boldsymbol{d}_g + \boldsymbol{\eta}_3)
\end{aligned}$$

$$
\begin{aligned}
\frac{\partial \boldsymbol{M}_3}{\partial k_3} =& -\frac{1}{2k_3^2}\boldsymbol{\eta}_3^T\boldsymbol{\eta}_3 + \frac{1}{2k_3^2}(k_3\boldsymbol{U}\boldsymbol{d}_g+\boldsymbol{\eta}_3)^T\left(\boldsymbol{I}_p + k_3\boldsymbol{U}\boldsymbol{U}^T\right)^{-1}(k_3\boldsymbol{U}\boldsymbol{d}_g+\boldsymbol{\eta}_3) \\
& -\frac{1}{k_3}(\boldsymbol{U}\boldsymbol{d}_g)^T(\boldsymbol{I}_p + k_3\boldsymbol{U}\boldsymbol{U}^T)^{-1}(k_3\boldsymbol{U}\boldsymbol{d}_g+\boldsymbol{\eta}_3) \\
& +\frac{1}{2k_3}(k_3\boldsymbol{U}\boldsymbol{d}_g+\boldsymbol{\eta}_3)^T(\boldsymbol{I}_p + k_3\boldsymbol{U}\boldsymbol{U}^T)^{-1}\boldsymbol{U}\boldsymbol{U}^T(\boldsymbol{I}_p + k_3\boldsymbol{U}\boldsymbol{U}^T)^{-1}(k_3\boldsymbol{U}\boldsymbol{d}_g+\boldsymbol{\eta}_3)
\end{aligned}
$$

F Initial parameter estimates

We first run *limma* to get false discovery rate (FDR) adjusted p-value of moderated t-test for each gene transcript. We then partition the gene transcripts to 3 clusters based on the FDR-adjusted p-values and signs of the test statistics: (1) gene transcripts with positive test statistics and FDR-adjusted p-values < 0.05 (denoted as OE); (2) gene transcripts with negative test statistics and FDR-adjusted p-values < 0.05 (denoted as UE); and (3) the remaining gene transcripts (denoted as NE). If numbers of transcripts in OE and/or UE are smaller than 5, we then use 100 transcripts having the smallest un-adjusted p-values to find initial OE and UE transcripts. We next estimate the cluster mixture proportions (π_1, π_2, π_3) by the 3 cluster sizes dividing the total number of gene transcripts.

Within each cluster, we use moment estimator to get the initial estimates of model parameters. In our case, we use sample median (med) and sample median absolute deviation (mad) to get robust estimates of mean and standard deviation, based on which we get the initial estimate of $\boldsymbol{\psi}$.

F.1 OE gene transcripts

$$
\begin{aligned}
\hat{\tau}_g^{(0)} =& \frac{1}{(\text{mad}_l(d_{gl}))^2} \\
\hat{\alpha}_1^{(0)} =& \left(\frac{\text{med}_g(\hat{\tau}_g^{(0)})}{\text{mad}_g(\hat{\tau}_g^{(0)})}\right)^2 \\
\hat{\beta}_1^{(0)} =& \frac{\text{med}_g(\hat{\tau}_g^{(0)})}{\left(\text{mad}_g(\hat{\tau}_g^{(0)})\right)^2} \\
\hat{\boldsymbol{\eta}}_1^{(0)} =& \left(\boldsymbol{W}_{(p+1)\times n}\boldsymbol{W}^T_{n\times(p+1)}\right)^{-1}\boldsymbol{W} log\left(|\hat{\boldsymbol{\mu}}|\right) \\
\hat{k}_1 =& \bar{\hat{\tau}}_g^{(0)} s^2.
\end{aligned}
$$

where

$$
s^2 = \frac{1}{n}\sum_{l=1}^{n}\left[\hat{\mu}_l - \bar{\hat{\mu}}\right]^2, \bar{\hat{\mu}} = \frac{1}{n}\sum_{l=1}^{n}\hat{\mu}_l, \bar{\hat{\tau}}_g^{(0)} = \frac{1}{G_1}\sum_{g=1}^{G_1}\hat{\tau}_g^{(0)}.
$$

F.2 UE gene transcripts

$$\hat{\tau}_g^{(0)} = \frac{1}{\left(\text{mad}_l(d_{gl})\right)^2}$$

$$\hat{\alpha}_2^{(0)} = \left(\frac{\text{med}_g(\hat{\tau}_g^{(0)})}{\text{mad}_g(\hat{\tau}_g^{(0)})}\right)^2$$

$$\hat{\beta}_2^{(0)} = \frac{\text{med}_g(\hat{\tau}_g^{(0)})}{\left(\text{mad}_g(\hat{\tau}_g^{(0)})\right)^2}$$

$$\hat{\boldsymbol{\eta}}_2^{(0)} = \left(\boldsymbol{W}_{(p+1)\times n}\boldsymbol{W}^T_{n\times(p+1)}\right)^{-1}\boldsymbol{W}log\left(|-\hat{\boldsymbol{\mu}}|\right)$$

$$\hat{k}_2 = \bar{\hat{\tau}}_g^{(0)} s^2.$$

where

$$s^2 = \frac{1}{n}\sum_{l=1}^{n}\left[\hat{\mu}_l - \bar{\hat{\mu}}\right]^2, \bar{\hat{\mu}} = \frac{1}{n}\sum_{l=1}^{n}\hat{\mu}_l, \bar{\hat{\tau}}_g^{(0)} = \frac{1}{G_2}\sum_{g=1}^{G_2}\hat{\tau}_g^{(0)}.$$

Here, we use $|-\hat{\boldsymbol{\mu}}|$ to make sure it is positive.

F.3 NE gene transcripts

The initial values are

$$\hat{\tau}_g^{(0)} = \frac{1}{\left(\text{mad}_l(d_{gl})\right)^2}$$

$$\hat{\alpha}_3^{(0)} = \left(\frac{\text{med}_g(\hat{\tau}_g^{(0)})}{\text{mad}_g(\hat{\tau}_g^{(0)})}\right)^2$$

$$\hat{\beta}_3^{(0)} = \frac{\text{med}_g(\hat{\tau}_g^{(0)})}{\left(\text{med}_g(\hat{\tau}_g^{(0)})\right)^2}$$

As for $\boldsymbol{\eta}_3$, we first need to get $\hat{\boldsymbol{\theta}}_g$. With linear regression, we have

$$\hat{\boldsymbol{\theta}}_g = \left(\boldsymbol{U}_{p\times n}\boldsymbol{U}^T_{p\times n}\right)^{-1}\boldsymbol{U}_{p\times n}\boldsymbol{d}_g$$

Then we can get

$$\hat{\boldsymbol{\eta}}_3^{(0)} = \text{med}_g\left(\hat{\boldsymbol{\theta}}_g\right)$$

And

$$\hat{k}_3 = \bar{\hat{\tau}}_g^{(0)} s^2$$

where

$$s^2 = \frac{1}{G_3}\sum_{l=1}^{G_3}\left(\hat{\boldsymbol{\theta}}_g - \bar{\hat{\boldsymbol{\theta}}}\right)^T\left(\hat{\boldsymbol{\theta}}_g - \bar{\hat{\boldsymbol{\theta}}}\right), \bar{\hat{\boldsymbol{\theta}}} = \frac{1}{G_3}\sum_{g=1}^{G_3}\left(\hat{\boldsymbol{\theta}}_g\right), \bar{\hat{\tau}}_g^{(0)} = \frac{1}{G_3}\sum_{g=1}^{G_3}\hat{\tau}_g^{(0)}.$$

G Convergence criterion

We stop the expectation-maximization iterations based on a proportional change, i.e. if the max of the absolute value of the differences of model parameter estimates between current iteration and previous iteration over the absolute value of the previous iteration estimates is smaller than a small constant (e.g. $1.0 \times$ 10-3).

Note that *eLNNpairedCov* and *eLNNpairedCov.SEM* can report empty OE and/or empty UE if it did not detect any over-expressed and/or under-expressed genes.

H Bounds for hyper-parameters

Although we can re-parameterize hyper-parameters to make sure $\alpha_c > 0$, $\beta_c > 0$, $0 < k_c < 1$, $c = 1, 2, 3$, and to transform constrained optimization problem to un-constrained optimization problem, the results are usually not good due to non-linear optimization in the M-step of the EM algorithm. To avoid unexpected large values of estimated α_c, β_c, and/or k_c in numerical optimization, we set constraints $0.001 < \alpha_c < 6$, $0.001 < \beta_c < 6$, $0.001 < k_c < 0.9999$.

If initial estimates $\hat{\alpha}_c$, $\hat{\beta}_c$, or $\hat{k}_c$ are outside their ranges, then we randomly choose a number between the range as the initial parameter estimates.

In the M-step of the EM iterations, we also require each element of $\boldsymbol{\eta}_c$, $c = 1, 2, 3$, be within the interval $[-10, 10]$.

If at least one element of $\boldsymbol{\psi}^{(t)}$ are outside their ranges in the t-th iteration of the EM algorithm, we then set final estimate of $\boldsymbol{\psi}$ as $\boldsymbol{\psi}^{(t-1)}$, and then only update $\pi_c^{(t-1)}$, $c = 1, 2, 3$, until convergence criterion satisfied.

The M-step in the EM algorithm is to maximize Q function, which is a nonlinear function of model parameters π_1, π_2, π_3, $\boldsymbol{\psi}$. It is difficult to optimize nonlinear functions. The above constraints and procedure produced good results in the real and simulation data analyses in this article.

I Information about Supplementary Tables for differentially expressed transcripts for GSE24742

The information about the differentially expressed gene transcripts detected by *limma*, *eLNNpairedCov* and *eLNNpairedCov.SEM* is shown in the 5 csv files: Table S1.csv − Table S5.csv.

The legends of these 5 Supplementary Tables are shown in the following table (Table A1).

Table A1: Legends of Supplementary Tables

**Table Name**	**Table legends**
Table S1	Gene list of 6 UE transcripts detected by *limma*
Table S2	Gene list of 55 OE transcripts detected by *eLNNpairedCov*
Table S3	Gene list of 59 OE transcripts detected by *eLNNpairedCov.SEM*
Table S4	Gene list of 355 UE transcripts detected by *eLNNpairedCov*
Table S5	Gene list of 352 UE transcripts detected by *eLNNpairedCov.SEM*

References

[1] Petersen, KB and Pedersen, MS. The matrix cookbook. https://www.math.uwaterloo.ca/~hwolkowi/matrixcookbook.pdf, 2012.
